# Oral Language Deficits in Familial Dyslexia: A Meta-Analysis and Review

**DOI:** 10.1037/bul0000037

**Published:** 2016-01-04

**Authors:** Margaret J. Snowling, Monica Melby-Lervåg

**Affiliations:** 1 Department of Experimental Psychology, University of Oxford; 2 Department of Special Needs Education, University of Oslo

**Keywords:** dyslexia, endophenotype, family risk of dyslexia, language impairment, multiple risk factors

## Abstract

This article reviews 95 publications (based on 21 independent samples) that have examined children at family risk of reading disorders. We report that children at family risk of dyslexia experience delayed language development as infants and toddlers. In the preschool period, they have significant difficulties in phonological processes as well as with broader language skills and in acquiring the foundations of decoding skill (letter knowledge, phonological awareness and rapid automatized naming [RAN]). Findings are mixed with regard to auditory and visual perception: they do not appear subject to slow motor development, but lack of control for comorbidities confounds interpretation. Longitudinal studies of outcomes show that children at family risk who go on to fulfil criteria for dyslexia have more severe impairments in preschool language than those who are defined as normal readers, but the latter group do less well than controls. Similarly at school age, family risk of dyslexia is associated with significantly poor phonological awareness and literacy skills. Although there is no strong evidence that children at family risk are brought up in an environment that differs significantly from that of controls, their parents tend to have lower educational levels and read less frequently to themselves. Together, the findings suggest that a phonological processing deficit can be conceptualized as an endophenotype of dyslexia that increases the continuous risk of reading difficulties; in turn its impact may be moderated by protective factors.

Literacy skills are the key to educational attainments and these in turn promote access to career opportunities and employment. However, even in developed educational systems, a substantial number of children fail to learn to read to age-expected levels. A specific barrier to progress is difficulty in decoding print—the first step to reading with understanding; such difficulties with the development of decoding skills are referred to as dyslexia. Dyslexia is a common condition, thought to affect some 3–7% of the English-speaking population, with more boys than girls affected (Rutter et al., 2004).

The definition of dyslexia has undergone many changes over the years, not least because there are no clear diagnostic criteria (Rose, 2009). Rather the distribution of reading skills in the population is continuous and the “cut-offs” used to define a person as “dyslexic” are arbitrary (e.g., Pennington, 2006). Moreover, dyslexia tends to co-occur with other disorders, including specific language impairment (SLI; e.g., Bishop & Snowling, 2004; McArthur, Hogben, Edwards, Heath, & Mengler, 2000; Melby-Lervåg & Lervåg, 2012), speech sound disorder (e.g., Pennington & Bishop, 2009), and attention-deficit/hyperactivity disorder (ADHD; e.g., McGrath et al., 2011; Willcutt & Pennington, 2000). Consistent with this, the Diagnostic and Statistical Manual of the American Psychiatric Association (*Diagnostic and Statistical Manual for Mental Disorders–Fifth Edition*, *DSM–5*; American Psychiatric Association, 2013) groups together reading disorders (dyslexia), mathematical disorders, and disorders of written expression under a single overarching diagnosis of Specific Learning Disorder within the broader category of Neurodevelopmental Disorders.

It has been known for many years that dyslexia, like other neurodevelopmental disorders, runs in families and studies of large twin samples demonstrate that reading and the phonological skills that underpin it are highly heritable (Olson, 2011, for a review). However, the genetic mechanisms involved are complex and depend on the combined influence of many genes of small effect and gene—gene interactions (e.g., Newbury, Monaco, & Paracchini, 2014). In addition, “generalist” genes, thought to code for general cognitive abilities, contribute to the etiology of dyslexia (Plomin & Kovas, 2005). Arguably, progress in the neurobiology of dyslexia requires a refined understanding of the phenotype of dyslexia at the cognitive level which clarifies its key developmental features and its relationship to other disorders. The current review contributes to that understanding.

In this article we provide a comprehensive review of studies that, assuming the heritability of dyslexia, have compared children at family risk (FR) of dyslexia with children with no such risk (low risk, control) on behavioral measures of perception, language, and cognition. Such studies have typically been designed to test out causal hypotheses regarding the developmental course of dyslexia. The methodology has distinct advantages over the more standard case-control approach; first it is free of clinical bias because children are recruited before they enter formal reading instruction; second, it allows us to disentangle possible causes of dyslexia from its consequences; third, and arguably most importantly, it is the only methodology that can allow the identification of risk factors in the preschool period, together with likely protective factors. Although such longitudinal prediction can also be conducted using a general population sample, the high-risk method is much more efficient; for instance, in a general population sample with a rate of dyslexia at 10%, one would need to follow 500 children to obtain a sample of 50 dyslexics; in a high-risk sample with a rate of dyslexia around 30%, a sample of 150 will yield the same number of affected cases.

The current review is organized around the main questions that family risk studies have addressed: the prevalence of dyslexia in children with a first-degree-affected relative, the nature of the home literacy environment in dyslexic families and the possible causes of dyslexia. We use the findings to address two further important issues for theory and practice. First, the relationship between dyslexia (diagnosed when written language skills fail to develop adequately) and language impairment (diagnosed when spoken language skills fail to develop adequately; Bishop & Snowling, 2004; Ramus et al., 2012). Second, evidence regarding what works to prevent or ameliorate dyslexia in children at high risk of the disorder. We begin with a short review of the research that guided our hypotheses before outlining the methodology for the current study and the constructs that were evaluated in the meta-analysis.

## Individual Differences in Reading Development

To consider the nature and developmental course of dyslexia, it is important to highlight the resource demands of learning to read. Universally, reading is a process of mapping between the visual symbols on the page (orthography) and the spoken language. However, the nature of the symbols and the units of spoken language to which they connect differ across languages (e.g., Ziegler & Goswami, 2005). In an alphabetic language such as English, the foundation of literacy is a system of mappings between letters and phonemes (the smallest speech sounds of words) and a challenge for the child is to abstract the mapping principle (Byrne, 1998). In contrast, Chinese is nonalphabetic; the orthographic symbols are characters comprising semantic and phonetic radicals that correspond to morphemes (units of meaning). The semantic radical provides information about the meaning and the phonetic radical provides a cue to the pronunciation of the word (Shu & Anderson, 1997).

Orthographies also differ in their “transparency”—that is the regularity of the mappings between symbols and sounds. Among European languages, English has the most inconsistent writing system, embodying many exceptions; German and Dutch have relatively few inconsistencies and Finnish is the most consistent. In general, it has been shown that regular languages pose fewer challenges for the beginning learner than irregular languages (Seymour, 2005) but arguably, this is a simplistic view. Languages also differ in the way in which they convey the grammar in writing and differences in morphological structure may moderate the ease of learning (Seidenberg, 2011).

Nonetheless, it is clear that the specific demands of learning to read will differ across languages. Irrespective of this, the child needs to learn the symbol set and how that set maps to the language and this requires explicit awareness of the structure of the language. Once the basic mappings have been established, reading practice (“print exposure”) is required to achieve reading fluency.

Among alphabetic languages it is well established that the predictors of individual differences in decoding (the skill that is impaired in dyslexia) are the same regardless of the transparency of the language: these are, letter knowledge, phoneme awareness, and rapid automatized naming (Caravolas et al., 2012; Ziegler et al., 2010a). However, reading development proceeds faster in the more transparent orthographies than in opaque orthographies (Caravolas et al., 2013). The process of learning is more protracted in languages that have large symbol sets and in nonalphabetic languages where mappings are to meaning rather than sound (Nag, Caravolas, & Snowling, 2011). Nonetheless, a similar set of skills appear to predict progress in these languages—namely symbol knowledge, metalinguistic awareness, and rapid automatized naming; the corollary is that deficits in these skills compromise decoding in dyslexia.

## The Role of Environmental Factors

The majority of research on dyslexia has focused on its proximal cognitive causes; however, within a developmental framework it is important also to consider distal risk factors. Given the differences between orthographies, one extrinsic factor which affects the rate of reading development is the language of learning. Thus, it might be expected that the prevalence of dyslexia will depend on the transparency of the language; indeed it has sometimes been speculated that the difficulty of the English language could be a cause of dyslexia. However, data from more regular orthographies provide little evidence in support of this contention (for Dutch: Blomert, 2005; for German: Fischbach et al., 2013).

Social-demographic factors can also affect reading attainments. For example, higher rates of reading difficulty have been reported in inner-city samples than in rural areas (except in developing countries where the opposite trend is seen) and it is well-known that there is a social gradient in reading attainment such that reading is poorer in disadvantaged groups (e.g., Rutter & Maughan, 2005). Among the factors that could account for demographic variation are differences in parental education level (Phillips & Lonigan, 2005, for a review) or in quality of schooling, although whether such differences are truly environmental, rather than reflecting genetic factors, is a moot point (Friend, DeFries, & Olson, 2008). The quality of the home literacy environment (e.g., Frijters, Barron, & Brunello, 2000) is one factor that likely reflects the correlated effects of genes and environment (*ge* correlation). Moreover, evidence suggests that the home literacy environment may at least partially mediate the influence of socioeconomic status (SES) on children’s literacy outcomes (e.g., Chazan-Cohen et al., 2009).

Home literacy environment is defined by a range of factors concerning parents’ and children’s attitudes and dispositions toward reading, including how long parents spend reading with their children and parents’ own reading behavior. An important component is “shared book reading” that has a significant impact on children’s oral language development (Bus, van Ijzendoorn, & Pellegrini, 1995). In addition, direct teaching of literacy concepts is observed in some families and this strengthens the foundational skills for reading. More important, differences in parental attitude to literacy appear to affect how children’s reading skills develop: while direct instructional practices focusing on print concepts facilitate the early development of decoding skills, shared language experiences around books have a greater impact on reading comprehension (Senechal & LeFevre, 2001). An important question that arises from this research is how the literacy environment in families in which there is a parent (or indeed a child) with dyslexia differs from that in a family where family members are free of literacy problems. Family risk studies provide the opportunity to do this and to track possible changes in both child- and parental attitudes as reading develops over time.

## Perceptual and Cognitive Deficits in Dyslexia

A major thrust of research on dyslexia has been to specify the underlying deficits that are candidate causes of the condition. Such impairments (that may be cognitive and/or have their origins in basic perceptual processes) mediate the impact of heritable, brain-based differences on behavior (Morton & Frith, 1995; Pennington, 2002; Ramus, 2003, 2004).

Within this approach, the predominant view for many years was that dyslexia could be traced to deficits within the phonological system of language (Melby-Lervåg, Lyster, & Hulme, 2012; Vellutino, Fletcher, Snowling, & Scanlon, 2004). As we have seen, phonological skills are critical foundations for learning to read in alphabetic systems. More generally, phonological deficits have been reported to characterize dyslexia in logographic Chinese (Hanley, 2005; Ho, Chan, Lee, Tsang, & Luan, 2004; Ho, Chan, Tsang, & Lee, 2002) and poor readers of alphasyllabic scripts (Nag & Snowling, 2011). However, a problem in assessing the causal status of phonological deficits is that performance on phonological tasks (such as phoneme awareness and nonword repetition) is influenced by reading skill (Morais & Kolinsky, 2005 for a review). It follows that deficits in these processes could be correlates (rather than causes) of poor reading. An advantage of family risk studies is that phonological processing can be measured before the onset of literacy. As we shall see, all family risk studies have assessed the development of phonological skills.

At a more fine-grained level, research has pursued the causes of the phonological deficits in dyslexia. In now classic work, Tallal (1980) proposed that dyslexia is caused by a problem with the rapid temporal processing of auditory information needed for the perception of speech sounds, leading to a cascade of difficulty from auditory processing through speech perception to phonological skills. Family risk studies are well placed to test such causal chains from the early stages of language development. Accordingly many of these studies draw on research suggesting deficits in speech perception in dyslexia (e.g., Adlard & Hazan, 1998; Nittrouer, 1999; Serniclaes, Van Heghe, Mousty, Carré, & Sprenger-Charolles, 2004; Ziegler, Pech-Georgel, George, & Lorenzi, 2009) or in basic auditory processing (Hämäläinen, Salminen, & Leppänen, 2012 for a review).

A separate line of investigation has focused on possible causes of difficulties with the letter-by-letter structure of words (orthographic deficits) in dyslexia. One theory is that spatial coding deficits affect ocular motor control (Boden & Giaschi, 2007; Kevan & Pammer, 2008; Vidyasagar & Pammer, 2010). Alternatively, problems in the system of visual attention could affect the left-to-right extraction of orthographic information critical for parsing letter strings before decoding (Facoetti, Paganoni, Turatto, Marzola, & Mascetti, 2000; Valdois, Bosse, & Tainturier, 2004). In addition, there are modality—general theories that aim not to explain particular features of dyslexia but rather seek overarching explanations (e.g., Ahissar, Lubin, Putter-Katz, & Bani, 2006; Nicolson & Fawcett, 1990; Vicari, Marotta, Menghini, Molinari, & Petrosini, 2003). Whereas such theories may hold promise for understanding how dyslexia relates to other co-occurring disorders (comorbidity, e.g., Rochelle & Talcott, 2006) they do not explain why dyslexia can and sometimes does occur in the absence of any other cognitive deficits.

Nevertheless, as a long history of the search for subtypes of dyslexia attests, these causal hypotheses are not mutually exclusive and it is important to recognize that dyslexia is a heterogeneous condition (e.g., Ramus et al., 2003). As Pennington (2006) has argued, the etiology of complex disorders like dyslexia is multifactorial and involves the interactions of risk and protective factors. Longitudinal studies of children at family risk of dyslexia that follow children from early childhood to formal schooling can reveal the risk factors associated with a dyslexia outcome. In addition, because dyslexia is a dimensional disorder, the study of unaffected relatives can be informative in highlighting protective or compensatory factors that mitigate familial risks. Such risks (that could be biological processes or cognitive impairments) can be described as “endophenotypes” (Skuse, 2001).

According to Bearden and Freimer (2006), an endophenotype is a “marker” that is associated with the disorder in the population and expressed at a higher rate in unaffected relatives of probands than in the general population. Put another way, it is intermediate between the genotype and the phenotype and, importantly, the impact of such processes can be moderated or compensated for by areas of skill or through interventions. This particular characteristic of an endophenotype deserves mention in relation to comorbidities. When two neurodevelopmental disorders frequently co-occur it is probable that they have endophenotypes in common (Thapar & Rutter, 2015, for a review). Prospective family risk studies can identify putative endophenotypes of dyslexia (or subclinical features) that mark the presence of co-occurring disorders For example, findings of family risk studies can elucidate relationships between difficulties with oral language observed in preschool and later written language disorders, in short, the comorbidity between dyslexia, specific language impairment (Bishop & Snowling, 2004; Pennington & Bishop, 2009, for reviews).

## Study Rationale and Hypotheses

Although the discussion above highlights the importance of taking a longitudinal perspective to understanding dyslexia, current knowledge of dyslexia draws mainly on cross-sectional studies involving the comparison of individuals with dyslexia and controls at one specific point in time. Such evidence cannot distinguish adequately between causal and noncausal reasons for associations. Following the pioneering work of Scarborough (1989, 1990), who followed a group of 2-year-old English-speaking children deemed to be at high-risk of dyslexia because of an affected parent, many prospective studies of children at family risk of dyslexia have begun in recent years. At the time of writing, 21 independent studies have been completed, resulting in 95 behavioral publications that are the focus of this review. In addition, our review found 23 descriptive studies using neurophysiological measures that are listed in Supplemental Material Table S1 (in the online supplement file). We used the findings of the behavioral studies to test the hypotheses that follow.

*Hypothesis 1:* The prevalence of dyslexia in children at family risk. We predicted that dyslexia would be more common in at-risk than control families (Hypothesis 1a) and more prevalent in English because it has an opaque orthography than in other European languages (Hypothesis 1b); in addition, given the large character set, we expected a high prevalence in Chinese. Irrespective of orthography, because dyslexia is a dimensional disorder with no clear boundaries, we predicted that prevalence would depend upon the cut-off used for “diagnosis” (Hypothesis 1c).

*Hypothesis 2:* The home and literacy environment of children at family risk of dyslexia. Because of the influence of genetic factors and the environments correlated with them, we hypothesized that the home literacy environment would differ between families in which there is a history of reading difficulty from that in families where parents are free of such problems (Hypothesis 2a). We did not specify the ways in which these differences would be manifest but we anticipated that parents with dyslexia would read less for pleasure than controls (Hypothesis 2b). If the home literacy environment influences children’s reading attainment, we predicted that parental literacy skills (a proxy for gene—environment correlation; van Bergen, van der Leij, & de Jong, 2014) would account for independent variance in children’s reading outcomes (Hypothesis 2c).

*Hypothesis 3:* Endophenotypes of dyslexia. Although endophenotypes can take many forms, we are concerned here with the perceptual and cognitive deficits that are observed among children at family risk of dyslexia. These can be expected to be present in the preschool years before dyslexia is diagnosed (where they can be construed as cognitive risk factors; Hypothesis 3a). Based on the overlap between dyslexia and language impairment (Bishop & Snowling, 2004; Ramus et al., 2012), we expected that delays and difficulties with speech and language development would be common (Hypothesis 3b). More specifically, for alphabetic languages we predicted that three of the skills considered foundational for literacy (letter knowledge, phoneme awareness, and rapid automatized naming [RAN]; Caravolas et al., 2013) would show developmental delay in the preschool period (Hypothesis 3c) and deficits in each would characterize dyslexia in the school years (Hypothesis 3d). Given the known continuity of risk for dyslexia among family members (Pennington & Lefly, 2001), we predicted that unaffected children at family risk of dyslexia would also have poor literacy skills relative to controls but not severe enough to warrant a diagnosis of dyslexia (Hypothesis 3e) and deficits/endophenotypes should be observed in unaffected children but to a milder degree (Hypothesis 3f). Finally, we aimed to evaluate the causal status of basic deficits in speech perception, visual, and auditory processing, attention and motor skills as additional risk factors (Hypothesis 3a).

*Hypothesis 4:* Predictive relationships between early cognitive abilities and later reading. Reading builds upon spoken language and there are similar heritable influences on both reading comprehension and language comprehension (e.g., Keenan, Betjemann, Wadsworth, DeFries, & Olson, 2006). In this light, we predicted that preschool measures of oral language would predict later reading outcomes, particularly reading comprehension (Hypothesis 4a). Based on previous findings (e.g., Caravolas et al., 2013) we also predicted that, for alphabetic languages, there would be three predictors of decoding skills and hence dyslexia: phonological awareness, symbol knowledge (letters in alphabetic languages) and RAN (Hypothesis 4b).

*Hypothesis 5:* Interventions for dyslexia. Arguably the ultimate aim of research on dyslexia is to identify effective interventions that will ameliorate its impact on educational attainments. In addition, training studies are important theoretically as tests of causal hypotheses (Snowling & Hulme, 2011). We expected that interventions incorporating training in letter knowledge and phoneme awareness would improve decoding skills in children at family risk of dyslexia (Hypothesis 5a). However, we did not expect such interventions to impact reading comprehension beyond gains in decoding unless they incorporated training in broader oral language skills (e.g., vocabulary training; Hypothesis 5b).

### Structure of the Current Review

Table 1 presents a summary of the hypotheses that guided the review. First, to assess Hypotheses 1(a–c) we summarize data from studies examining prevalence of dyslexia in family risk studies; second, to assess Hypotheses 2 (a and b), we use data on the home and literacy environment and to assess Hypothesis 2c we use data examining parental skills as predictors of outcome. To assess evidence for endophenotypes (Hypotheses 3 a–f), we adopt a developmental perspective, presenting evidence from different developmental stages from infancy/toddlerhood to secondary school. Here we will discuss findings of studies that have compared children at family risk of dyslexia with controls (at low risk because they do not have a family history). These studies can tell us about the risk factors associated with familial dyslexia (Hypotheses 3a–d). We will also include findings from retrospective analyses comparing the cognitive profiles of children at family risk who have later been classified as dyslexic or not, and controls. In addition to reinforcing the conclusions above, these studies can assess Hypothesis 3e and elucidate endophenotypes of dyslexia (Hypothesis 3f). Next we will examine the findings of longitudinal studies that have incorporated multiple regression and related statistical techniques to provide additional evidence regarding the predictors of dyslexia (Hypotheses 4a and b) and finally evaluate the evidence for effective interventions (Hypothesis 5a and b).[Table-anchor tbl1]

## Method

The review was designed and is reported in line with the Preferred Reporting Items for Systematic Reviews and Meta-Analyses (PRISMA) statement (www.prisma-statement.org). PRISMA is a consensus statement developed by an international group of researchers in health care for the conduct and reporting of systematic reviews and meta-analyses. When there is a sufficient number of studies (two or more), we will do a meta-analysis by calculating a mean effect size. If a summary effect is not possible, we will do a systematic review and report effect sizes for individual studies.

### Literature Search, Inclusion Criteria, and Coding

#### Literature search

**Figure 1 fig1:**
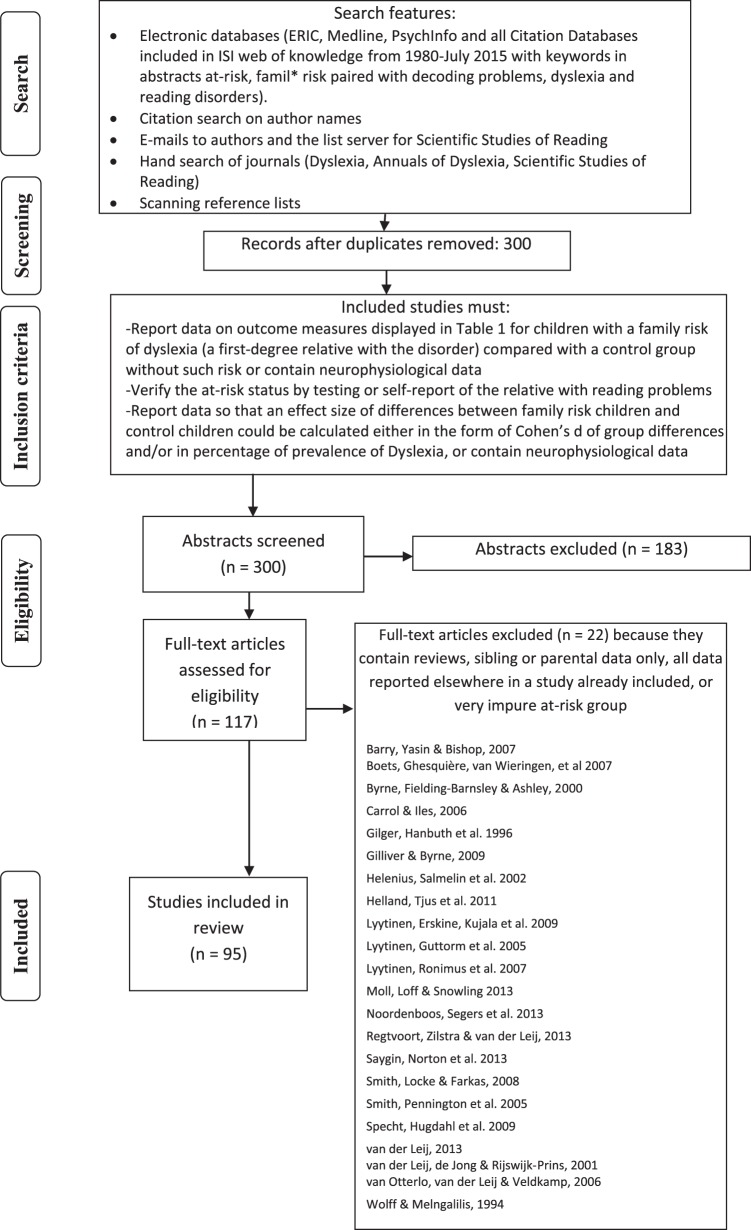
Flow diagram for the search and inclusion criteria for studies in this review. Adapted from “Preferred Reporting Items for Systematic Reviews and Meta-Analyses: The PRISMA Statement,” by D. Moher, A. Liberati, J. Tetzlaff, D. G. Altman, and The PRISMA Group, 2009. *PLoS Med 6*(6). Copyright, 2009 by the Public Library of Science.

Details concerning the literature search, criteria for inclusion and flow of studies are shown in Figure 1. The literature search consisted of the following components: Electronic databases (ERIC, Medline, PsychInfo, and all Citation Databases included in ISI web of knowledge from 1980–30 July 2015 with keywords in abstracts at-risk, famil* risk paired with dyslexia, reading disorders, decoding, and decoding problems), citation search on author names, emails to authors and posting at the list server for Scientific Studies of Reading, hand search of journals (Dyslexia, Annuals of Dyslexia, Scientific Studies of Reading), scanning reference lists, and Google Scholar.[Fig-anchor fig1]

#### Inclusion criteria

To be included a study had to consist of a sample of children with familial risk of dyslexia. Family risk was defined as having a parent and or a sibling (a first-degree relative) with dyslexia. The at-risk status had to be verified by testing or self-report of the relative with reading problems. In addition, the studies had to include a control group of children with no known family risk of dyslexia. Studies vary in the nomenclature they have used. To be consistent, in this review, we use the terms “FR–Dyslexia” to refer to children at family risk (FR) who are identified as having reading problems, “FR–NR” to refer to children at family risk who are identified as typical in their literacy outcome (normal reader) and “control” to refer to children from low-risk groups with no family history, who are considered free of reading difficulties.

In the review we focus on three different study designs: (a) group comparisons, (b) longitudinal prediction studies, and (c) experimental intervention studies. We also include references to neurophysiological studies that are tabulated in Table S1 in the online supplement file. The studies included had to report data so that an effect size could be calculated or significance testing reported for either one of three types of comparisons, based on the different designs we found in the studies in this field: (Comparison Type 1) between children at family risk of dyslexia (FR+) and children without such risk (control). In these studies, reading skills are treated as a continuous variable, and family risk children are not separated into two groups based on whether they have dyslexia or not; (Comparison Type 2) between children at family risk later “diagnosed” with dyslexia (FR–Dyslexia) and control children without family risk (control); and (Comparison Type 3) children at family risk who did not fulfil criteria for dyslexia (FR–NR) and control children without family risk (control).

For the longitudinal prediction studies, in addition to the criteria outlined above, the study must have reported a longitudinal analysis concerning either (a) unique predictors of outcomes treated as continuous variables, or (b) unique predictors of literacy outcome when this is treated as a categorical variable (i.e., presence or absence of reading problem). For a study to be included, the analysis needs to have been conducted so that the predictive patterns pertaining to children at family risk and children without such risk could be compared, or the effect of group membership could be determined. For the experimental intervention studies, some kind of training must have been included with the aim of ameliorating difficulties of reading or literacy in children at family risk.

The neurophysiological studies in Table S1 in the online supplement file used a variety of methodologies and were not amenable to meta-analysis. Most of these studies are of small samples; however, their findings will be used when appropriate to reinforce conclusions.

#### Coding

Some of the at-risk studies are longitudinal and report data from different stages in development. When coding the studies, it became clear that although there are 95 different publications, many were based on the same study sample (there are 21 independent samples). In Appendices A, B, and C (Column 1 in parentheses after the author name), the sample on which the publication is based is indicated. Special attention had to be taken in the coding to avoid bias related to dependency in the data. To make use of as much information as possible from the different publications, information was coded for different developmental stages: (a) Infants and toddlers (below the age of 3 years), (b) Preschool (below 5.5 years and before formal reading instruction starts), (c) Early Primary school (up to 4th grade), and (d) Late primary school/secondary school (from 5th grade and upward). By doing this, we were able to code information from longitudinal studies twice without merging data from the same study in the same analysis. Therefore, we did not violate the assumption of independence in the data. Nonetheless effect sizes in the different developmental stages will be related because 20 out of 69 publications included in the analyses of group comparisons have data coded from more than one developmental stage.

Furthermore, scrutiny of the studies revealed that several different measures were often reported for the same construct, sometimes in different publications. Table 2 presents the indicators that we selected to represent the higher-order constructs in the review. If there was more than one indicator for a construct from the same developmental stage (e.g., Boston naming test and word definitions for vocabulary knowledge), either in the same or in different publications, the mean of the indicators coded was used in the analysis. An advantage of this procedure is that the mean effect size will be more reliable because it is based on two measures of the same construct. The number of studies and sum of participants reported for the different analyses refers to the number of independent samples and participants that have provided data on a construct and the number of effect sizes contributing to a mean effect size is reported in parentheses. However, if the same test was reported in several different publications on the same sample, the same test was only coded once. In many cases, prevalence of dyslexia is reported for the same sample in several publications. However, such data were only coded once for each independent sample based on one set of reading tests in each study. When it was unclear whether data were based on the same sample or parts of the same sample, authors were contacted by email and asked for clarification. Most authors responded, but in cases where they did not, we have based the overview on information provided in the articles.[Table-anchor tbl2]

A random sample of 80% of the studies was coded by two independent raters. The interrater correlation (Pearson’s) for main outcomes (means, *SD*, sample size, and age) was *r* = .98 and agreement rate = 93%. Any disagreements between raters were resolved by consulting the original article or by discussion.

### Procedure and Analysis

The coding of studies and analyses were conducted using the “comprehensive meta-analysis” program (Borenstein, Hedges, Higgins, & Rothstein, 2005). Two different effect sizes were used in the meta-analysis. For group differences between the family risk groups and the children from low-risk groups with no family history, we used Cohen’s *d*. Cohen’s *d*s were calculated using Hedges’ corrections for small sample sizes (Hedges & Olkin, 1985). When Cohen’s *d* is negative, children from high-risk groups with a family history have the lowest score. For judging the size of the effect, for group comparisons Cohen’s tentative guidelines were used. According to Cohen, *d* = 0.2 is a small effect, 0.4 is moderate and 0.8 is large. However, note that according to Cohen (1968), such guidelines should only be used when no better basis for estimating the effect size is available. In the intervention studies, if Cohen’s *d* is positive, the group that has received the intervention has the highest gain between pre- and posttest. For the intervention studies, two influential policy organizations (What works clearinghouse [WWC] and Promising Practices Network [PPN]), have set a limit of *d* = 0.25 for when results of high-quality randomised trials should be taken as having policy implications (see Cooper, 2008). In the intervention studies we adopt these guidelines in preference to Cohen. For estimates of prevalence of children in the family risk samples and the control samples affected with dyslexia, we used percentage affected with dyslexia as the effect size.

Mean effect sizes were estimated by calculating a weighted average of individual effect sizes using a random effects model. A mean effect size was calculated if there were two or more studies. A 95% confidence interval (CI) was calculated for each effect size to establish whether it was statistically significantly larger than zero. If confidence intervals cross zero, the result is not significantly different from zero.

To examine the variation in effect sizes between studies, the *Tau*^*2*^ was used (Hedges & Olkin, 1985). As a rule of thumb, if *Tau*^*2*^ exceeds 1, the variation between the studies is large. *I*^2^ was also used to determine the degree of true heterogeneity. *I*^*2*^ assesses the percentage of between-study variance that is attributable to true heterogeneity rather than random error. Notably, *I*^*2*^ does not say anything about the size of the variation between the studies in general, that is, *I*^*2*^ can be 100% or 10% but in both cases variation between studies can be small or large. Thus, *I*^*2*^ can only be used to determine the part of the heterogeneity that is due to true variation between studies rather than sampling error.

For moderator variables, studies were separated into subsets based on the categories in the categorical moderator variable, and a *Q*-test was used to examine whether the effect sizes differed between subsets. When there were fewer than two studies in a category, this analysis was not conducted. To examine the size of difference between subsets of studies, overlap between CIs was also examined. In cases of multiple significance tests of moderators on the same data set, the results are reported both with and without Bonferroni correction.

When we coded articles, it became clear that there were numerous instances of missing data. If data were critical to calculate an effect size, articles with missing data were excluded if authors did not respond to an email request to provide the data (see inclusion criteria in flowchart). In cases where an effect size could be computed on one outcome but data were missing on other outcomes or moderator variables, the study was included in all the analyses for which sufficient data were provided.

### Results From the Review

Appendices A, B, and C contains all the necessary information to replicate the results from our meta-analysis. Appendix A shows characteristics of studies comparing children at family risk (not yet diagnosed) and control children. In the results, data based on Appendix A are presented as group comparisons of children at family risk versus controls not at-risk. Appendix B shows characteristics of studies comparing children at family risk diagnosed with dyslexia and controls and the prevalence of dyslexia. In the results, data based on Appendix B are presented as group comparisons of (a) family risk children with dyslexia versus controls not at-risk and (b) family risk children who are normal readers versus controls not at-risk. Appendix C shows characteristics of intervention studies concerning children with family risk of dyslexia; results based on these data are presented in a separate section of the review on intervention studies.

### Prevalence of Dyslexia

#### Prevalence of dyslexia in family risk samples

**Figure 2 fig2:**
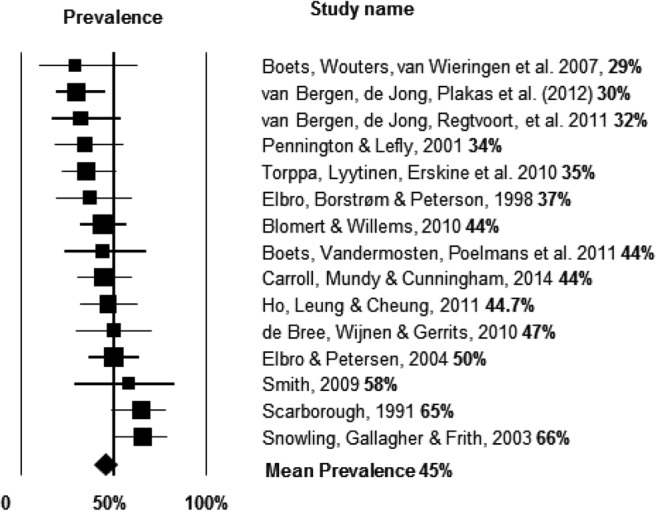
Prevalence of dyslexia in the family risk samples (mean prevalence across all studies [displayed by ♦] and prevalence for each study [displayed by ■] with 95% confidence intervals presented by vertical lines).

The first analysis considered the prevalence of dyslexia in families where there was a positive history. Fifteen independent studies had examined prevalence of dyslexia/reading disorders in samples of children at family risk. The studies included 420 children with dyslexia (mean sample size 29.4, *SD* = 13.9, range 9 to 53). Mean prevalence (expressed as percentage) of dyslexia in the family risk samples in the different studies was 45% (95% CI [39%, 51.2%] *I*^2^ = 36.5%, *Tau*^*2*^ = 0.08; see Figure 2), confirming that children with a first-degree relative with reading problems have a high risk of developing such problems themselves^1^ (Hypothesis 1a). [Fig-anchor fig2]

An analysis of moderator variables showed that, as to be expected, the cut-off point for diagnosing dyslexia had a significant impact on the mean prevalence (Hypothesis 1c). In eight studies where the criterion for diagnosing reading disorder was set above the 10th percentile, the mean prevalence was 53% (95% CI [46.3%, 60.0%], *I*^2^ = 13.54%, *Tau*^*2*^ = 0.02) while in six studies where the criterion was set below the 10th percentile, the mean prevalence was 33.7%, (95% CI [26.6%, 41.8%], *I*^2^ = 0%, *Tau*^*2*^ = 0). Notably, one study was excluded from this analysis because it did not report a cut-off. Because of distributional characteristics, the impact from the diagnostic cut-off variable had to be separated into two categories rather than be analyzed as a continuous variable; the difference between the two categories was significant, *Q*(1) = 11.84, *p* < .001, also after correcting for multiple comparisons, *p* = .001.

Turning to differences in prevalence according to orthography (excluding Chinese studies), for English 54% (95% CI [41.3%, 66.2%], *I*^2^ = 52.85%, *Tau*^*2*^ = 0.17). For studies from more transparent languages (Dutch, Danish, or Finnish), 40% (95% CI [35.0%, 46.4%], *I*^2^ = 0%, *Tau*^*2*^ = 0). This difference was approaching significance, *Q*(1) = 3.62, *p* = .058. Further, after examining the studies, it was clear that more studies with English samples had lenient cut-off criteria. After controlling for cut-off in the analysis of group differences in orthography, the difference between the groups was not significant (β = −0.21, *p* = .13; Hypothesis 1b).

#### Prevalence of dyslexia in control samples

To estimate how much the risk of dyslexia is increased in families with a history of reading difficulty, it is important also to assess the prevalence of dyslexia in control samples without a family history. Eleven studies reported information from the control group; the studies included 540 control children without family risk (mean sample size 49.09, *SD* = 20.5, range 23 to 93). The mean prevalence of dyslexia in the samples without family risk was 11.6% (95% CI [8.4%, 15.8%] *I*^2^ = 35.23%, *Tau*^*2*^ = 0.12). Compared with the 45% prevalence of dyslexia found in the family risk group, this confirms that the children at family risk have a reliably higher likelihood of developing dyslexia/reading disorders than children in the general population without such risk (there is no overlap between CIs; Hypothesis 1a).

In six studies where the criterion for diagnosing reading disorder was set above the 10th percentile, the mean prevalence was 16% (95% CI [11.5%, 21.2%], *I*^2^ = 6.97%, *Tau*^*2*^ = 0.02) while in four studies where the criterion was set below the 10th percentile, the mean prevalence was 7.8% (95% CI [4.9%, 12.2%], *I*^2^ = 0%, *Tau*^*2*^ = 0). One study did not report the cut-off. The difference between the two categories was significant, *Q*(1) = 4.09, *p* = .01, also after correcting for multiple comparisons (*p* = .02). As for differences between orthography, for the English studies,11.2% (95% CI [0.06%, 19.8%], *I*^2^ = 46.41%, *Tau*^*2*^ = 0.21); for studies from more transparent languages, 11.6% (95% CI [7.6%, 17.2%], *I*^2^ = 38.44%, *Tau*^*2*^ = 15). This difference was not significant, *Q*(1) = 0.46, *p* = .50. Finally, the single study reporting prevalence in a family risk study of Chinese readers estimated this to be 47% but no data were reported for controls.

### The Home and Literacy Environment of Children at Family Risk of Dyslexia

A small number of family risk studies have explored the hypothesis that parents with dyslexia offer a different “diet” of books, print, and other literary experiences to parents who have not experienced reading problems.

#### Parental education

One factor which might influence the home literacy environment is parental educational level. Four studies comparing children at family risk (FR+) with controls report data on the education level of the mothers. In these studies, the difference in mothers’ educational levels was small and not significant, *d* = 0.13 (95% CI [−0.32, 0.07], *Tau*^*2*^ = 0.00, *I*^2^ = 0). Three studies reported data on fathers’ education, and here the difference between the FR+ and control groups was larger, *d* = −0.63 (95% CI [−1.43, 0.17], *Tau*^*2*^ = 0.45, *I* = 90.75%). One study (Black, Tanaka, et al., 2012) also reported differences in parental verbal language skills (as measured by WAIS verbal IQ), finding that FR+ mothers had significantly poorer verbal skills than those without such risk *d* = −0.73, (95% CI [−1.20, −0.17]). As for fathers, the difference was smaller and not significant, *d* = −0.37 (95% CI [−0.91, 0.18]).

Stronger evidence comes from three studies that compared groups according to children’s literacy outcomes. Mothers of children with a reading disorder (FR–Dyslexia) had moderately lower education levels than mothers of controls, but this difference is not significant, *d* = −0.32 (95% CI [−2.06, 1.42], *Tau*^*2*^ = 2.18, *I* = 93.09%). A similar pattern is present when comparing mothers of children in the FR–NR group and controls, *d* = −0.68 (95% CI [−1.15, 0.20], *Tau*^*2*^ = 0.44, *I* = 76.44%). Compared with controls, fathers in the FR–Dyslexia group have moderately but not significantly lower levels of education, *d* = −0.45 (95% CI [−1.96, 1.06], *Tau*^*2*^ = 1.07, *I* = 89.78%). Fathers in the FR–NR group also have moderately lower educational level than fathers of controls and again the difference is significant, *d* = −0.47 (95% CI [−0.90, −0.04], *Tau*^*2*^ = 0, *I* = 0%, *k* = 2).

#### Home literacy environment

Torppa et al. (2007) made a comprehensive assessment of the home literacy environment (HLE) of families participating in the Finnish Jyväskylä Study. Parental questionnaires completed when children were 2, 4, 5, and 6 years of age provided measures of shared reading, access to print, the child’s interest in reading, and the modeling of literate behaviours in the home. The group means across age for the HLE composites were similar in children at family risk (FR+) and controls for shared reading across age groups, *d* = −0.08 (95% CI [−0.23, 0.05], *Tau*^*2*^ = 0, *I* = 0%); for access to print, *d* = −0.05, 95% CI [−0.20, 0.11], *Tau*^*2*^ = 0, *I* = 0%; and for child’s reading interest, *d* = −0.14, (95% CI [−0.28, 0.01], *Tau*^*2*^ = 0, *I* = 0%). However, the scores for shared reading when the children were 2 years old and for parental modeling of literate behaviors were more variable in the FR group (Hypothesis 2a). Moreover, there was a significant difference between the FR+ and control groups in the frequency of parental modeling activities in reading books, newspapers, magazines, and so forth, with parents from at-risk families typically engaging in such activities less frequently than parents without a history of dyslexia: for fathers, *d* = −0.44, (95% CI [−0.73, −0.16]) and for mothers, *d* = −0.66 (95% CI [−0.95, −0.38]; Hypothesis 2b).

Scarborough (1991b) also reported differences in literacy experiences of children from families at risk of dyslexia but data are not reported to allow calculation of an effect size. A graph presented in the article shows that during the preschool years, children who went on to be dyslexic (FR–Dyslexia) were read to less often by their fathers than children from the family risk group who went on to be normal readers and controls. For mothers, there was less joint reading for children at family risk at 30 months than for controls but not thereafter (Hypothesis 2a). However, mothers also rated how often they observed their children reading to themselves. There were fewer occurrences of solitary book reading by the FR–Dyslexia group at all ages (two to three times per week compared with five to seven times for controls) suggesting that group differences may be child-driven and less frequent exposure to books could be self-imposed by affected children. A follow-up in adolescence of children at family risk of dyslexia by Snowling, Muter, and Carroll (2007) found that there was a trend for those in the FR–Dyslexia group to report themselves as reading less than those in the FR–NR group, but this was not significant (*p* = .08). There were no group differences in how often the families bought books and gave books for presents (*p* = .25) but the dyslexia group had less knowledge of book titles and authors (but not magazines) as assessed by a print exposure questionnaire.

Exploring whether parental reading skills predict reading outcomes in children at family risk of dyslexia, (Torppa, Eklund, et al., 2011; *N* family risk dyads = 31, control dyads = 68) showed that beyond the influence of children’s language and decoding-related skills, parental literacy skills predicted early reading and spelling outcomes (Hypothesis 2c). However, parental skills did not account for variance in reading fluency in third grade once children’s skills at earlier points in time were taken into account.^2^ A similar conclusion was reached by Aro, Poikkeus et al. (2009) using data from the same study.

### Endophenotypes of Dyslexia

Having considered what is known about environmental variables, we turn to consider how being at family risk of dyslexia affects the development of perceptual and cognitive skills and ultimately literacy outcomes.

#### Characteristics of infants and toddlers at family risk of dyslexia

The first developmental stage investigated has been from birth to age 3, namely infants and toddlers. We will review data pertaining to each of the main constructs in turn to evaluate Hypotheses 3a and 3b.

Table 3 shows results from studies of infants and toddlers. First, note that the evidence is scarce. As can be seen in Table 3, there was a significant difference between family risk children who went on to develop dyslexia and control children in general cognitive abilities. Note that the test of cognitive abilities also included language-related tasks. Furthermore, the family risk children showed poorer articulatory skills than peers with no family risk, and also reliably poorer vocabulary knowledge and grammar. This was not the case for the family risk children who did not develop dyslexia (Hypothesis 3b).[Table-anchor tbl3]

A number of studies not amenable to meta-analysis have reported qualitative differences in language development between FR+ children and controls. An early study by Locke et al., (1997) suggested differences in babbling between children at family risk and controls. Also in infancy, Wilsenach and Wijnen (2004) demonstrated a preference for grammatical passages (as opposed to nongrammatical) containing forms of morphosyntactic agreement in Dutch among not-at-risk compared to at-risk infants, and van Alphen, de Bree et al. (2004; *N* family risk 35, controls 27) found that FR+ children paid reliably less attention to stress patterns in words than children not at-risk. Koster et al., (2005; *N* family risk = 111, 87 controls) focused on the growth of vocabulary. For common nouns and predicates, FR+ and typically developing (TD) groups followed the same course but there was divergence for the production of verbs and closed class words when the FR+ group appeared to plateau at a vocabulary size between 50 and 100 words. These findings chime with those of Scarborough (1990, 1991a) who reported group differences in lexical diversity, accuracy of phonological production, utterance length, and grammatical complexity in natural language samples. Finally, Lyytinen, Poikkeus, Laasko, Eklund, and Lyytinen (2001; *N* = 107 FR+ and 93 controls) documented the emergence of language skills in children at family risk of dyslexia and controls. Group differences did not emerge until 24 months when the family risk group used shorter sentences. Whereas late talkers in the control group tended to catch up, those in the family risk group remained delayed up until 3.5 years.

Finally, six small-scale neurophysiological studies (Table S1 in the online supplement file) reported differences in brain response (primarily ERP) to speech stimuli and two studies reported reduced sensitivity to higher word-level semantic information in infants at family risk of dyslexia.

#### Characteristics of preschool children at family risk of dyslexia

The studies reviewed in this section concern preschool children, aged from 3–5.5 years (dependent on school entry in the country of sample origin). Table 4, Panel A, B, and C shows the results from these studies. The focus is on the known precursors of word decoding as well as more general developmental progress (Hypotheses 3a and b).[Table-anchor tbl4]

##### General cognitive abilities and perceptual skills

Table 4 Panel A shows mean effect size with 95% CIs, sample size (studies, effect sizes, and persons) and heterogeneity measures for measures of general cognitive abilities, perceptual processes, and motor skills. For nonverbal IQ, it is apparent that groups differ more in the studies comparing FR+ children and controls, than in the studies in which the family risk group with and without dyslexia are separately compared with controls.

There are few studies of auditory or visual processing in this age group. For auditory processing, there is a significant difference in studies that compare FR+ children and controls, and in one longitudinal study, children at family risk who go on to be dyslexic (FR–Dyslexia) demonstrate poorer auditory processing skills than controls but the FR–NR group do not differ significantly (Hypothesis 3a). In addition one neurophysiological study reports reduced sensitivity to rapid auditory processing. Likewise studies of speech perception are inconclusive and data are not amenable to meta-analysis: studies from the Utrecht dyslexia project reported differences between children at family risk of dyslexia and controls in categorical speech perception for consonants (Gerrits & de Bree, 2009), but not for a vowel continuum (van Alphen, et al., 2004). From the same project, de Bree, van Alphen, Fikkert, and Wijnen, (2008) assessed comprehension of metrical stress using pictures of objects with stress patterns which were either strong**–**weak (′zebra) or weak–strong (ka′do), accompanied by presentations of the object name pronounced with correct or incorrect stress. Overall, FR+ children looked relatively less to the targets when they heard a weak—strong pattern than controls. In addition one neurophysiological study reports reduced sensitivity to phoneme deviance. For neither visual processing nor motor skills are there reliable group differences, but the evidence here is extremely limited with very few studies.

##### Oral language skills

Table 4 Panel B shows mean effect size with 95% CIs, sample size (studies and persons) and heterogeneity measures for speech and language skills including articulatory accuracy, vocabulary knowledge, grammar, phonological memory, and verbal short-term memory (STM; Hypothesis 3b). For articulatory accuracy, there are few studies of preschool children so the evidence is limited. Across three concurrent studies, FR+ children show reliably poorer articulatory accuracy than controls. However, group differences are not significant when the family risk group is classified according to reading outcome. One of the longitudinal samples overlaps with one of the concurrent studies examining FR+ children. Further, two studies found that, although 3-year-old children at family risk of dyslexia did less well on speech production tasks than controls, their errors obeyed Dutch stress rules (de Bree, Wijnen, & Zonneveld, 2006; Gerrits & de Bree, 2009).

For lexical/vocabulary knowledge, children at family risk have reliably poorer skills than controls. The results show that the FR–Dyslexia group shows a large deficit in vocabulary knowledge in preschool. The FR–NR group also shows a significant deficit, but in size this is only one-quarter of the size compared with the effect size for those who do develop a reading problem. Here, one sample included as a concurrent comparison is also included in comparisons comparing FR–Dyslexia and FR–NR groups with controls. For grammar, children at family risk have reliably poorer grammatical skills than their peers, irrespective of whether they actually go on to develop a reading problem. However, those who develop reading problems have more severe difficulties (Hypothesis 3f).

Turning to measures of verbal/phonological processing, children at family risk have significantly poorer phonological memory than controls for all comparison types (there is one overlapping sample between the different comparisons). FR+ children also have reliably poorer verbal STM than controls; those who go on to have reading problems (FR–Dyslexia) have particularly severe difficulties whereas the FR–NR group does not differ reliably from controls. In this case, there are three overlapping samples between the comparison types and the differences between the family risk and control groups increase when publication bias is taken into account.

##### Decoding-related skills

Table 4 Panel C shows mean effect size with 95% CIs, sample size (studies and persons) and heterogeneity measures for decoding-related predictors, that is, letter knowledge, phoneme awareness, rhyme awareness and rapid naming (Hypothesis 3c). First, for letter knowledge, it is clear that FR+ children and family risk children who go on to develop a reading problem are slow to acquire letter knowledge. The FR–NR group is also reliably slower to acquire letter knowledge than the controls, but the effect size is only one third compared with the FR+ children. It is noteworthy that effect sizes decline for letter knowledge when publication bias is taken into account; also three samples overlap between the group comparisons.

For phoneme awareness, there is a marked deficit in preschool children at family risk, but this is larger for those who go on to develop a reading problem (FR–Dyslexia). The group difference between FR–NR and control groups is barely significant, and drops to nonsignificant when adjusted for publication bias. Three comparisons involve overlapping samples.

For rhyme awareness the evidence is very limited, but there are reliable differences both between FR+ and control groups, and between FR–Dyslexia and control groups. For rapid naming, all three comparisons suggest reliably poorer skills in family risk children regardless of outcome and controls. However, performance on rapid naming tasks is much poorer in the FR–Dyslexia than the FR–NR groups, as shown by the confidence intervals which show little overlap. Furthermore, the differences between these two comparisons increases when publication bias is taken into account. There are two overlapping samples between the comparisons.

For phoneme awareness it is possible to do a moderator analysis to examine the effect of orthography in studies comparing children at family risk with controls. The results for English studies was *d* = −0.42, 95% CI [−0.64, −0.20], *p* = .0001, *Q*(2) = 1.39, *p* > .01, *Tau*^*2*^ = 0, *I* = 0%, *k* = 3 and for studies from more transparent languages (Danish, Finnish, and Italian) *d* = −0.60, 95% CI [−0.76, −0.43], *p* = .0001, *Q*(3) = 0.52, *p* > .01, *Tau*^*2*^ = 0, *I* = 0%, *k* = 4. This difference was not significant, *Q*(1) = 1.59, *p* = .21.

#### Characteristics of children in early primary school at family risk of dyslexia

The studies reviewed in this section concern primary schoolchildren from school entry to 4th grade (age dependent on school entry in the country of sample origin). Table 5 Panel A, B, C and D shows the results from these studies.[Table-anchor tbl5]

##### General cognitive abilities and perceptual skills

Table 5 Panel A shows mean effect size with 95% CIs, sample size (studies and persons) and heterogeneity measures for skills related to general cognitive, perceptual and motor skills (Hypothesis 3a). For nonverbal IQ, there is a significant difference in nonverbal IQ between FR+ and control groups, with no overlap between the samples in the three comparison types. For auditory processing, visual processing and motor skills, the evidence is very limited. There are, however, reliable differences in auditory and visual processing, but not in motor skills between family risk and control groups. In addition, two neurophysiological studies report deviant auditory processing in children at family risk of dyslexia.

##### Oral language skills

Table 5 Panel B shows mean effect size with 95% CIs, sample size (studies and persons) and heterogeneity measures for oral language skills. For articulatory accuracy, vocabulary knowledge, and grammar, the evidence suggests that the difficulties observed early in development amongst children at family risk of dyslexia in these areas are mostly resolved in early primary school. An exception seems to be vocabulary knowledge, where the FR–Dyslexia group still have reliably poorer skills than their peers (Hypothesis 3a). For phonological and verbal STM, results indicate that the problems here are more severe and persistent and poor verbal STM is observed in the FR+ as well as the FR–Dyslexia groups (Hypothesis 3a). While the number of studies here are limited, there is no overlap between the samples in the different comparison types.

##### Decoding-related skills

Table 5 Panel C shows mean effect size with 95% CIs, sample size (studies and persons) and heterogeneity measures for decoding-related predictors. For letter knowledge, the difficulties experienced in preschool seem to have resolved by early primary school. However, problems with phoneme awareness are now more severe both in FR+ children and in the FR–Dyslexia group (Hypothesis 3d). For the FR–NR group, the problems are less severe, but the group difference is still as much as half a *SD* unit (Hypothesis 3f). This is not the case for rhyme awareness: here both FR+ children and the FR–Dyslexia group show poor performance but the FR–NR group do not differ from controls. Similarly, for rapid naming, the FR–Dyslexia group has persistent and severe difficulties, while those who do not develop a reading problem have no difficulties (FR–NR; Hypothesis 3d). For phoneme awareness one study is based on the same sample for all three comparison types, for rhyme awareness all comparison types are based on the same sample and for the other measures there is no overlap.

For phoneme awareness in studies that compared family risk children with controls it was possible to analyze orthography as a moderator. For English studies, *d* = −0.96 (95% CI [−1.64, −0.28], *p* = .03, *Tau*^*2*^ = 0.26, *I* = 72.82%) and for studies from more transparent languages, *d* = −0.72 (95% CI [−1.33, −0.10], *Tau*^*2*^ = 0.27, *I* = 71.72%). This difference was not significant, *Q*(1) = 0.27, *p* = .60.

##### Literacy skills

Table 5 Panel D shows mean effect size with 95% CIs, sample size (studies and persons) and heterogeneity measures for literacy outcomes namely word reading, nonword reading, spelling, and reading comprehension. For literacy outcomes, the FR–Dyslexia group has severe difficulties; their skills are around 2 *SD* units poorer than controls on all measures, implying a cut-off of around the 2nd percentile. Thus, even though the analysis of prevalence showed that the cut-off limits for reading disorder varied considerably, on average at a sample level, the reading disorders are severe. In addition, the FR–NR group have moderate difficulties in all literacy domains compared with the controls but with performance reliably better than that of the FR–Dyslexia group (Hypothesis 3e) except for reading comprehension where there was no overlap between the confidence intervals. For word decoding there are three overlapping samples between the comparisons, for nonword decoding one overlapping and for spelling three overlapping samples.

For orthography, a moderator analysis for studies examining word reading was possible for all three comparison types. For English studies comparing FR+ and control groups, *d* = −0.56 (95% CI [−0.98, −0.13], *Tau*^*2*^ = 0, *I* = 0%, *k* = 2) and for studies from more transparent languages *d* = −1.08 (95% CI [−1.65, −0.40], *Tau*^*2*^ = 0.33, *I* = 80.53%, k = 5). This difference was not significant, *Q*(1) = 2.04, *p* = .15. For the comparison of FR–Dyslexia and control groups in English studies, *d* = −2.68 (95% CI [−3.50, −1.86], *Tau*^*2*^ = 0.47, *I* = 73.33%, *k* = 4) and for studies from more transparent languages, *d* = −2.39 (95% CI [−2.84, −1.94], *Tau*^*2*^ = 0.0, *I* = 0%, *k* = 3). This difference was not significant, *Q*(1) = 0.37, *p* = .54. For studies comparing the FR–NR group with controls: for English, *d* = −0.41 (95% CI [−0.70, −13], *Tau*^*2*^ = 0, *I* = 0%, *k* = 4), and for studies from more transparent languages *d* = −0.28 (95% CI [−1.85, 0.07], *Tau*^*2*^ = 0.0, *I* = 0% *k* = 3). This difference was not significant, *Q*(1) = 0.36, *p* = .55.

#### Longer-term outcomes: Group comparisons in late primary and secondary school

The evidence for long-term outcomes of children at family risk is limited, and consists of two studies, Snowling, Muter, and Carroll (2007) and Elbro and Petersen (2004; reading comprehension). The results reported in these studies are summarized in Table 6. Table 6 suggests that children at family risk who develop dyslexia show persistent difficulties in a range of language- and literacy-related areas. Those family risk children who do not develop a reading disorder also experience some difficulties but these appear to be restricted to word-level reading and spelling (Hypothesis 3f).[Table-anchor tbl6]

### Longitudinal Prediction Studies

Thus far the review has highlighted some of the precursors of dyslexia. However, since many of these are correlated with each other, their causal status is unclear. Stronger evidence for causal risk factors comes from longitudinal studies that assess the factors that make a unique contribution to literacy outcomes or to dyslexia status as a categorical variable. It was not possible, given the diversity of the published longitudinal prediction studies and the fact that many report data on the same sample, to code a correlation matrix based on mean correlations from the studies and use this in a metaregression. Therefore, we present the findings study by study. We focus here on studies predicting literacy outcomes. We also detected four longitudinal studies (all based on the Jyväskylä sample) predicting vocabulary knowledge (Laakso, Poikkeus, & Lyytinen, 1999; Lyytinen, Ahonen, et al., 2001; Lyytinen et al., 2001; Natale, Aunola, et al., 2008). Because literacy was not an outcome in these studies, they were considered to be beyond the scope of this review.

The first longitudinal study to report analyses of the predictors of literacy outcome for at-risk and control samples separately was by Pennington and Lefly (2001; *N* family risk = 67, control = 57 studied from kindergarten to summer after second grade). Using stepwise multiple regression analysis they reported that for controls, the pattern of prediction was stable across age, with phonological awareness being the main predictor, accounting for between 18 and 39% of the variance in the literacy variables. In contrast, for the family risk group, the predictive pattern changed over time, with letter knowledge as the most important predictor at the first two time points and phonological awareness at Time 3 (Hypothesis 4b).

Using the same sample, Cardoso-Martins and Pennington (2004; same *N* as in the previous paragraph) assessed the contribution of phoneme awareness and rapid naming to literacy outcomes. For controls, none of the rapid naming measures contributed significantly to reading or spelling when the differences in children’s IQ and phoneme awareness were taken into account. However, for children at family risk, rapid naming skills were uniquely related to literacy skills (Hypothesis 4b). Together the findings suggest that the acquisition of phonological awareness is delayed in children at family risk of dyslexia and learning to read may be more dependent, at least initially, on letter knowledge.

The largest family risk study to date is the Jyväskylä study. Eight publications predicting literacy skills are based on this sample. Among these, a series of three have examined the relative influence of oral language and decoding-related skills on literacy outcomes (Hypothesis 4a and b). Torppa, Poikkeus et al. (2007; *N* family risk = 96, controls = 90) examined the development of phonological awareness and early reading, also taking account of the home literacy environment (age 6.5). Despite the FR+ group having poorer language skills, the predictive pattern was very similar for the two groups though, in the FR+ group, the home literacy variables and children’s reading interest had stronger associations with each other and with skill development. A latent variable model using growth curves showed that both for FR+ children and controls, vocabulary and letter knowledge predicted phonological awareness and vice versa, phonological awareness also predicted vocabulary and letter knowledge. The effects of home literacy environment, principally shared reading, were mediated by vocabulary. Taking this further, Torppa, Lyytinen et al. (2010; *N* FR–Dyslexia = 46, FR–NR = 68, control = 84) used a path model with observed variables to examine the longitudinal predictors of reading. There were strong predictive links to reading (particularly to reading accuracy) from receptive and expressive language (Hypothesis 4a), via letter knowledge, rapid naming, phonological awareness, and morphology (Hypothesis 4b; see also Torppa, Eklund, et al., 2011 above).

In addition one study has examined neurophysiological measures of speech perception as predictors of literacy skills (Pennala, Eklund, et al., 2010; *N* FR–Dyslexia = 35, FR–NR = 69, control = 80). Beyond STM, phonological memory and rapid naming, phonemic length discrimination ability in Grade 1 explained both spelling and reading skills as late as third grade (*R*^2^ reading accuracy = 15%, spelling 12%).

Another important contribution of the Jyväskylä group has been to describe trajectories of reading development and predictors of literacy development at the individual level. Torppa, Poikkeus et al. (2006; *N* family risk = 96, controls = 90) examined the development of letter knowledge using longitudinal trajectory analysis. Three separate clusters were identified to describe development: delayed, linearly growing, and precocious. The delayed group was dominated by children at family risk of dyslexia. Phonological sensitivity, phonological memory, and rapid naming significantly predicted letter knowledge, and there was a strong relationship between the letter knowledge cluster to which a child belonged and their early reading skills.

Developmental trajectories from birth to school age were also examined by Lyytinen, Erskine et al. (2006; *N* family risk = 106, controls 93). In this study, mixture modeling revealed four subgroups with different trajectories of development. The largest subgroup, labelled “typical trajectory,” contained mostly children from the control group. A group labelled “unexpected trajectory” contained an equal number at-risk and not-at-risk. Two smaller subgroups showed either a “dysfluent trajectory” or a “declining trajectory.” The troubled developmental pathways were characterized by problems in phonological awareness, rapid naming or letter knowledge. Examining individual risk of dyslexia, Puolakanaho, Ahonen et al. (2007; *N* FR–Dyslexia = 46, without reading disabilities = 152) used receiver operating curve plots and logistic regression models to investigate the key predictors of outcome from the ages of 3.5–5.5 years. Results showed that the models with family risk, letter knowledge, phonological awareness, and rapid naming provided prediction reliability as high as 0.80 (Hypothesis 4b).

One further publication (Aro, Poikkeus, et al., 2009, *N* family risk = 101, controls = 89) went beyond children’s cognitive skills to examine the effects of risk accumulation across different domains on cognitive, behavioral, and academic outcomes. For reading fluency, a hierarchical regression analysis showed that family risk of dyslexia (vs. no risk) added significant explanatory value (8%, *p* = .000), and that neurocognitive risks (measured by a composite of earlier measured language skills, phonological awareness, rapid naming, visuomotor skills, motor skills, and memory) added a large amount of explanatory value (20.7%, *p* = .000) while parental literacy levels did not add significant explanatory value (1.7%, *ns* [*p* value not reported]).

Finally, one publication reports longitudinal predictive analyses for Chinese children at family risk of dyslexia (Wong, McBride-Chang, et al., 2012; *N* at-risk dyslexia = 57, at-risk without reading disorders = 57, no control data for sample not at-risk reported). Using three logistic equation models, the best-fitting models for predicting individual risk of dyslexia included all print-related variables (rapid naming, character recognition, and letter identification), and gender but not family risk. It should be noted, however, that the at-risk sample consisted of children identified as “at risk” because of poor performance on language tests, and not according to family risk only.

### Intervention Studies

The findings from the longitudinal studies are, arguably, most useful for identifying the precursors of dyslexia; however, the only way to establish more conclusively whether a causal relationship exists is through the use of experimental training studies (Bradley & Bryant, 1983). Six intervention studies investigating children at family risk of dyslexia are summarized in Appendix C. Four have used interventions to promote the foundations of decoding (letter knowledge and phonological awareness training; Hypothesis 5a), one used dialogical reading targeting broader language skills (encouraging the child to be the “reader” of a shared book and talk about it), and one used a combination of training of phonological awareness and broader language skills (Hypothesis 5b). There is only one randomized study, the others are quasi-experiments with a control group and baseline measures.

Table 7 show results from intervention studies. The results show effects on letter knowledge, phonological awareness, and word decoding when compared with at risk controls, but no effects when compared with controls not at risk (Hypothesis 5a). If we look at the studies that have included a follow-up measure in first or second grade, the results are disappointing: for word reading, *d* = −0.22 (95% CI [−0.50, 0.06]; compared with controls at-risk), and for one study of nonword reading, *d* = −0.10 (95% CI [−0.44, 0.42]; also compared with controls at-risk). In summary, the findings from intervention studies hold promise for promoting phoneme awareness, letter knowledge and decoding at posttest immediately after the intervention, but are not very encouraging in terms of transfer to follow-up tests of reading or language skills. Together they suggest that gains may be short-lived and it is difficult once poor reading has set in, for at-risk readers to catch up with their peers.[Table-anchor tbl7]

## Discussion

Studies of children at family risk of dyslexia have been conducted in several different alphabetic languages and Chinese. The findings are important because they derive from prospective longitudinal studies that have recruited children before they have failed to learn to read and, therefore, are comparatively free of clinical bias. This review presents the first comprehensive analysis of data from such studies and incorporates a meta-analysis. It provides robust evidence concerning the precursors of dyslexia in preschool and the familial risk factors associated with poor literacy attainments. It also elucidates potentially heritable endophenotypes of dyslexia and protective factors that enable some family members to “compensate” to circumvent poor reading, while others succumb to literacy problems.

The findings of our review are novel and surprisingly consistent across languages: there is a heightened risk of dyslexia in families in which a first-degree relative is affected, such that children at high risk are four times more likely to succumb to reading problems than peers from families with no such risk. A universal finding is that children at family risk of dyslexia acquire language more slowly than their peers, and signs of dyslexia are evident from preschool onward. Further it is evident that children at family risk of dyslexia are slow to develop phonological awareness and to learn letters (and symbols) such that, by early primary school, most children at family risk of dyslexia, and more specifically those who develop a reading problem, have difficulties in acquiring the skills that underpin these abilities, such as phoneme awareness.

### Summary of the Evidence

In the beginning of the article we laid out five sets of hypotheses. We now turn now to assess the evidence in support of each of the predictions with reference to these in Table 1.

#### 1. The prevalence of dyslexia in children at family risk

Using data from 15 studies we found evidence in support of Hypotheses 1a and c. Thus, the mean prevalence of dyslexia in children at family risk is 45%, with estimates ranging from 29%, for a Dutch study to 66% for an English study. Consistent with Hypothesis 1a, these estimates are much higher than those reported for control samples. Contrary to Hypothesis 1b, the effect of orthography was only marginal and the cut-off criterion for dyslexia was the only robust factor determining differences between studies. Indeed, consistent with Hypothesis 1c, the prevalence was lower for studies that used stricter (more conservative) criteria for classifying poor readers. Notwithstanding this, it was noteworthy that the average group deficit leading to a diagnosis of dyslexia was large across studies, suggesting the bottom 2% of readers were classified as dyslexic.

#### 2. Differences in the home and literacy environment of children at family risk of dyslexia and controls

Data on the home literacy environment of children at family risk of dyslexia are scarce. While it should be noted that many studies have deliberately recruited families to be matched for socioeconomic circumstances, we found evidence that provided some support for Hypotheses 2a, b, and c. Consistent with Hypothesis 2a, children at family risk appear to be read to less often than children in families in which neither parent is dyslexic (but it is important to bear in mind that this difference could be child-driven). Also as predicted by Hypothesis 2b, there was a trend for parents with dyslexia, perhaps unsurprisingly, to have lower educational levels and to read to themselves less frequently than parents of controls but in most cases differences fail to reach significance and are in need of replication. Such findings might explain why, consistent with Hypothesis 2c, parental reading status (itself possibly a proxy for gene—environment interaction) accounts for variance in the prediction of reading over and above what is predicted by cognitive variables

#### 3. Endophenotypes of dyslexia

Here we found evidence in support of all of our hypotheses. Consistent with Hypothesis 3a, there are very early developmental differences between family risk children who go on to be dyslexic and controls in general cognitive development. The limited evidence from preschool children suggests those at family risk of dyslexia have poor performance on auditory but not on visual processing tasks (though evidence for a direct causal influence of such skills on literacy outcomes is sparse).

As predicted by Hypothesis 3b, from infancy through toddlerhood, children at family risk of dyslexia who go on to develop dyslexia have reliably slower speech and language development as indicated by poorer performance than controls in tests of articulation, lexical/ vocabulary knowledge, and grammar. Children at family risk who do not go on to develop dyslexia show poorer performance on all measures than controls, but the size of the effects do not reach significance. In preschool, children at family risk of dyslexia are slow to develop language skills, with nonword repetition (phonological memory) skills being particularly poor. By school age however, apart from tasks related to phonological and verbal STM, many of the oral language problems evident in preschool seem to be resolved or are much milder by early primary school though children classified as dyslexic have significantly poorer vocabulary than controls (Hypothesis 3b).

During the preschool years, it is clear that children at family risk of dyslexia face challenges in acquiring letter knowledge, phoneme awareness and rapid naming skills, consistent with Hypothesis 3c. For rapid naming, nonword repetition and rhyme awareness family risk children who later develop a reading problem do particularly poorly but family risk children who go on to be normal readers also experience difficulties relative to controls, as they do in vocabulary, grammar, and letter knowledge (consistent with Hypothesis 3f). The two studies reporting findings for Chinese found deficits in knowledge of Chinese characters, phonological awareness of tones (paralleling letter knowledge and phoneme awareness in alphabetic languages), and in RAN.

In early primary school, family risk children in general (FR+), and more specifically those who develop a reading problem (FR–Dyslexia) have difficulties with decoding-related skills (Hypothesis 3d). Rhyme awareness and rapid naming show a different pattern than for the other predictors: children who do not develop a reading problem perform in the normal range in RAN tasks and in rhyme awareness. Consistent with Hypothesis 3e, family risk children who do not fulfil criteria for dyslexia (FR–NR) demonstrate reliably poorer performance than their peers in word decoding, nonword decoding and spelling underlining the fact that dyslexia is a continuous trait (e.g., Pennington, 2006). However, their deficits do not extend to reading comprehension (perhaps because this group has stronger vocabulary and language skills). In line with this view, children at family risk who do not develop reading problems appear to overcome delays in the development of vocabulary, grammar, and phonological skills observed in the preschool years by the time of formal schooling.

#### 4. Predictive relationships between early cognitive and later reading skills

The studies reviewed did not test our hypotheses directly but they do provide evidence largely consistent with both Hypotheses 4a and b. Results show that phonological awareness, letter knowledge and rapid naming are strong predictors of literacy skills in children at family risk of dyslexia, as they are in children without such risk. However, letter knowledge may be a predictor for a longer period of time in children at risk than in typical readers (where it reaches ceiling earlier) and hence rapid naming might be more important as a unique predictor in at-risk children. There is also evidence that parental reading status and shared book reading have an impact on children’s vocabulary and letter knowledge, and indirectly on literacy skills (Hypothesis 4b). Although there were not a sufficient number of studies to conduct a systematic analysis, a similar set of predictors (i.e., letter/symbol knowledge, phonological awareness, and rapid naming) stand out as important in English, Finnish, and Chinese children.

#### 5. Intervention studies

Studies that have evaluated interventions for dyslexia are generally poor in quality and evidence is, therefore limited. There is some evidence consistent with Hypothesis 5a that training in decoding related skills has positive effects but only when treated and untreated at-risk controls are compared. Regardless of the intervention, the size of the effects is small and the effects are not long-lasting. The evidence available neither confirms nor refutes Hypothesis 5b.

### Methodological Issues

This review has identified a number of methodological issues that limit the conclusions that can be drawn from studies of children at family risk of dyslexia. We used the GRADE system (Grading of Recommendations Assessment, Development, and Evaluation) to critically appraise the evidence that we have discussed. GRADE is an approach that rates the quality of evidence and is used widely by, for instance, Cochrane and the World Health Organization (Guyatt, Oxman, et al., 2008, 2011). Quality reflects confidence in the results based on the study limitations and risk of bias; there are four categories—high, moderate, low, and very low. Observational studies and intervention studies are rated based on the limitation of the study, inconsistency of results, indirectness of evidence, imprecision, and reporting bias. Here we will discuss the study quality of the observational studies under four criteria: failure to develop and apply appropriate eligibility criteria, flawed measurement, failure to control for confounding variables, and incomplete follow-up.

#### 1. Failure to develop and apply appropriate eligibility criteria

We found considerable variation across studies of children at family risk of dyslexia in how families are recruited: while most studies begin by recruiting children who have at least one parent with dyslexia, some have recruited younger siblings of children with dyslexia, and studies vary in how dyslexia is established in the index relative. Such variation may affect the identification of endophenotypes and affect conclusions regarding familial risk factors. For example, in many studies dyslexia status is based on self-report rather than confirmed with standardized tests and typically no screening is done for other conditions which may affect parents or other relatives, such as attention disorders (Snowling, Dawes, Nash, & Hulme, 2012). Further, parents who volunteer to take part in a study of children at family risk of dyslexia are already aware of the condition and, if diagnosed themselves, they will be highly motivated to ensure their children get the best of opportunities (see Leavett, Nash, & Snowling, 2014). In this light, the poor response of at-risk children to intervention may to some extent reflect that the home literacy environment of volunteers for such trials is already rich in activities to promote prereading skills.

#### 2. Flawed measurement

Not all of the studies which report group differences between family risk and no-risk groups have published data on dyslexia outcomes and, therefore, it is difficult to use these studies to clarify the precursors of dyslexia. Among those that have, sample sizes are often small. Furthermore, outcomes have not been defined consistently across studies with some studies assessing reading accuracy, speed, or fluency, others both accuracy and comprehension, and many silent with regard to IQ. Also a common limitation is that sensitivity and specificity data that could potentially add valuable information about the prospects of identifying dyslexia at an early age are not reported for the tests. More generally, measures, particularly of language, are not pure and often tap processes such as executive skills that are not controlled. Further, measurement error is rarely dealt with in the studies, and only a minority report α reliability, other types of measurement reliability or latent variables. The statistical treatment of data has also been diverse. While some studies use sophisticated multivariate techniques, most studies are relatively small given the parameters in the models and there has been a strong tendency to pool across high- and low-risk samples to examine the predictors of dyslexia. As a result the conclusions, particularly from predictive longitudinal and neurophysiological studies are in need of replication and should go beyond the investigation of predictors to examine the moderators or mediators of reading outcomes.

#### 3. Failure to control for confounding variables

A salutary finding of family risk studies is the lack of screening for comorbid disorders; undoubtedly this limits the conclusions that can be drawn. One possibility, which the extant studies are not set up to address, is that low general cognitive ability, a measure of basic processing capacity that draws on perceptual—motor as well as cognitive skills, is a marker of comorbid conditions. Notably, if samples of children at family risk of dyslexia contain some children with primary impairments of speech and language, this could explain why, at the group level, they show moderate to severe difficulties in the linguistic domain. Relevant to this issue, Nash, Hulme, Gooch, and Snowling (2013) reported that approximately one-third of their sample of children at family risk of dyslexia fulfilled diagnostic criteria for SLI. Further, two cross-sectional studies of children at family risk of dyslexia (FR+) provide some relevant data pertinent to the issue of uncontrolled speech problems (Carroll & Snowling, 2004; Carroll & Myers, 2010). In one of the Chinese studies reviewed, McBride-Chang et al. (2008) reported that 5-year olds with language-delay performed significantly worse than controls across all tasks known to be concurrent predictors of reading in Chinese (namely, syllable deletion, tone detection, RAN digits, morphological awareness, and a task requiring mapping of characters to sounds, tapping visuospatial processing). In contrast, children at family risk of dyslexia were only poorer in Chinese word recognition, tone detection and morphological awareness. In short, there is a need for research that tracks the growth of reading skills in children with different patterns of language strength and weakness as well as co-occurring risk factors (e.g., attention problems, McGrath et al., 2011) to identify pathways to dyslexia in children with a broad range of language learning impairments. Finally, another possible confounder that is rarely reported is gender. Although gender is sometimes seen as a risk/protective factor, very few studies report its effect on literacy outcomes.

#### 4. Incomplete follow-up

In many studies of children at family risk of dyslexia, especially those that are longer term, attrition rates are high, and there is little information about how the missing data have been dealt with.

The GRADE system lists five different study limitations that can afflict randomized controlled trials. However, only one intervention study with children at family risk was randomized. This is a major shortcoming of the extant research. In addition, although not discussed in the GRADE system but important to note, is the duplicate publishing of articles reporting data based on the same sample. When there are several publications based on the same sample it is critical to describe clearly the sample size and how it relates to previously documented research drawing on the same sample so that bias can be assessed.

In summary, the large and growing body of research on children at family risk of dyslexia has strengthened evidence for causal relationships between a range of oral language deficits and later literacy skills in this population. However, few studies have sampled from across the range of social classes, many have not matched groups for socioeconomic status and few have controlled for co-occurring disorders. An outstanding question is whether the effects of language are direct or fully mediated by skills which are the foundations of decoding and there is still a pressing need for higher quality training (experimental) studies.

### Conclusions and Educational Implications

Although there is debate as to whether dyslexia should be considered a diagnostic category, there is strong evidence from the studies reviewed here, that it is a developmental disorder, signs of which are apparent in the preschool years. This is the first rigorous review of the findings from the 21 studies that have followed children at family risk of dyslexia from preschool through the early school years, published in over 100 articles. Therefore, it provides a comprehensive analysis of the risks associated with familial dyslexia that predate the onset of literacy and elucidates likely causal factors. A novel finding is that the risks associated with dyslexia appear to be universal across languages. Because the focus was on perceptual and cognitive risk factors, the findings highlight potential targets for interventions to prevent dyslexia.

The findings of the review confirm that the likelihood of reading difficulties is significantly increased in children at family risk, with offspring in such families varying along a continuum of severity, consistent with the notion of a dimensional disorder. As for all complex disorders, an interaction of genetic and environmental risk and protective factors determine where the skills of an individual fall on this continuum. Although data are limited, studies of children at family risk of dyslexia also remind us of the importance of gene—environment correlations, captured for example, by differences, albeit subtle, in the home and literacy environment experienced by children of parents with dyslexia.

Turning to cognitive risk factors, studies of children at family risk confirm that a phonological deficit is a primary risk factor for dyslexia throughout development, consistent with findings from a large body of research on school-age children with dyslexia and universally across languages. In the preschool years this appears best captured by a deficit in phonological memory (nonword repetition) and in the school years by a deficit in phoneme awareness (that becomes more marked (relative to controls) in those with reading problems). However, it is clear that a phonological deficit alone is not sufficient to account for dyslexia (Pennington, 2006). Two findings are of particular importance. First, children at family risk who go on to be classified as “not dyslexic” share some of the same impairments as their affected relatives. Principally, they experience phonological difficulties, consistent with the notion of a “phonological endophenotype” of dyslexia (Moll, Loff, & Snowling, 2013). However, in preschool they also experience delayed language development, poorer letter knowledge and RAN deficits, relative to controls. Second, children who go on in the school years to be identified as dyslexic show deficits not only in phonological skills but also in broader language skills (grammar and vocabulary are typically affected in preschool and vocabulary knowledge during the school years). Additional risks are conferred by lower nonverbal ability, weaknesses in auditory processing and limitations of verbal STM. Together, these findings add to a growing body of evidence that a phonological deficit is one of a number of risk factors for dyslexia that accumulate toward a threshold that characterizes the disorder (Pennington et al., 2012).

In line with this, there is suggestive evidence that there is more than one developmental trajectory leading to dyslexia (e.g., Lyytinen et al., 2006). The findings of the review show that, although children at family risk of dyslexia share phonological deficits, not all become classified as poor readers. The divergence in the pattern of literacy development observed here is reminiscent of the pattern reported by Bishop and Adams (1990) for children with a preschool history of language difficulties who do and do not resolve their language impairments. Subgroup differences may turn on the severity of the phonological deficit—such that those with more severe deficits are more likely to be poor readers and have enduring vocabulary impairments. On the other hand, phonological deficits may have different origins in different subgroups; for example, as a consequence of poor verbal STM in children at family risk who do not develop reading problems or as a marker of language impairment and associated auditory processing deficits in children who go on to be dyslexic as a facet of language learning impairment (cf. Conti-Ramsden, Botting, & Faragher, 2001).

There are apparently also protective factors that prevent some children at family risk from developing significant reading difficulties. Such children tend to have stronger language skills (principally better vocabulary) and they perform within the normal range on rapid naming tasks. Good language skills may have both direct and indirect effects on reading development. According to Storch and Whitehurst (2001), the influence of language is indirect because children with better language have better phonological skills. Alternatively, Nation and Snowling (1998) proposed that children with decoding difficulties who have good language, particularly vocabulary, can make use of context to bootstrap their deficient skills. In this view, the influence of language is direct. Turning to RAN, again there are a number of possible interpretations. First, if RAN taps a brain mechanism or network involved in the cross-modal integration of visual (symbolic) and verbal codes as some have supposed (e.g., Lervåg & Hulme, 2009; Price & McCrory, 2005), then children who do well on such tasks will be better able to establish the mappings between orthography and phonology that are fundamental to reading. In this view, the protracted period of time required by children with dyslexia to learn letters (or other written symbols) is to be expected. Second, children with better RAN performance have faster speed of processing, indicative of greater resources that may, indirectly, help them to compensate. Indeed, RAN taps a variety of executive processes including sustained attention and inhibition (e.g., Clarke, Hulme, & Snowling, 2005) and children who are free of executive impairments are likely to learn to read better (e.g., Kegel & Bus, 2013).

Because risk and protective factors interact, preschool screening for dyslexia is not straightforward. Using data from the Jyväskylä study, Puolakanaho et al., (2007) showed that being at family risk of dyslexia increases the probability of reading disability. However, if letter-naming skills develop early, this decreases it dramatically. Similarly, for a child with poor letter-naming skills at 4.5 and 5.5 years, the probability of dyslexia is lower if he or she has good phonological awareness or efficient RAN skills. These findings represent a nuanced view of the etiology of dyslexia. First, it is in line with other sources of evidence showing that dyslexia is associated with multiple risks (Pennington et al., 2012; Snowling, 2008). Second, it highlights that different skills interact in the development of literacy, and therefore, dyslexia.

An important dilemma for practitioners is how to proceed in the knowledge that dyslexia is not a clear-cut diagnostic category but a dimensional disorder. This being the case, criteria for dyslexia need to be agreed externally and can be expected to differ in different contexts and in different developmental phases. This review shows that even individuals who do not reach criteria for dyslexia experience significant literacy problems relative to their peers; in situations where these difficulties are disabling, these individuals will need special arrangements. In terms of intervention, although there has been considerable progress in recent years in our understanding of methods for teaching reading (e.g., National Institute of Child Health and Human Development, 2000), effective interventions for those at family risk of dyslexia are awaited.

## Supplementary Material

10.1037/bul0000037.supp

## Figures and Tables

**Table 1 tbl1:** Hypotheses for the Review

Hypotheses guiding the review	Predictions
The prevalence of dyslexia in children at family risk	
Hypothesis 1a	Dyslexia is more common in at-risk than control families.
Hypothesis 1b	Dyslexia is more prevalent in English than in other European (regular) languages.
Hypothesis 1c	The prevalence rates for dyslexia depend upon the cut-off used for “diagnosis.”
The home and literacy environment of children at family risk of dyslexia	
Hypothesis 2a	The home literacy environment will differ between families in which there is a history of reading difficulty from that in families where both parents are free of problems.
Hypothesis 2b	Parents with dyslexia will read less for pleasure than controls.
Hypothesis 2c	Parental literacy skills will account for independent variance in children’s reading outcomes.
Endophenotypes of dyslexia	
Hypothesis 3a	Cognitive and perceptual risk factors that are putative endophenotypes should be present in the preschool years before dyslexia is diagnosed.
Hypothesis 3b	Children at family risk of dyslexia will show delayed speech/language development.
Hypothesis 3c	The development of three critical foundations for decoding, viz phonological awareness, symbol knowledge (letters in alphabetic languages) and rapid naming (RAN) will be delayed in children at family risk of dyslexia in preschool.
Hypothesis 3d	Deficits in phonological awareness, symbol knowledge and RAN will characterize dyslexia in the school years.
Hypothesis 3e	Unaffected children at family risk of dyslexia will have poorer literacy skills than controls.
Hypothesis 3f	Endophenotypes of dyslexia will be observed in unaffected children but to a milder degree than in affected children.
The predictive relationships between early cognitive and later reading skills	
Hypothesis 4a	Oral language skills will predict literacy outcomes in children at family risk of dyslexia.
Hypothesis 4b	Three critical foundations for decoding, viz phonological awareness, symbol knowledge (letters in alphabetic languages) and rapid naming (RAN) will be predictors of decoding in children at family risk of dyslexia.
Interventions for dyslexia	
Hypothesis 5a	Training in letter knowledge and phoneme awareness will ameliorate decoding difficulties.
Hypothesis 5b	Training in vocabulary and broader language skills will improve reading comprehension.

**Table 2 tbl2:** Indicators for the Constructs Targeted in the Review

Higher order constructs	Lower order constructs	Indicators coded
Home literacy environment	Parental education	Reports of educational background
	Book reading habits and availability	Various questionnaires and self-reports
	Parental literacy skills	Tests of parental language and reading skills
General cognitive abilities and perceptual skills	Nonverbal IQ	Nonverbal problem-solving tasks such as Raven, WISC performance, TONI
	Motor skills	Tests or rating scales of motor skills
	Auditory processing	Speech perception, tone perception, tone in noise detection
	Visual processing	Tests of visual perception (e.g. Gardner), visual matching, visual cueing
Oral language skills	Articulatory accuracy	Percentage of consonants correct, percentage of intelligible speech
	Vocabulary knowledge	Defining words, naming pictures, parental scales such as CDI
		Pointing at pictures (such as PPVT)
	Grammar	Plural marking, Past tense test, temporal terms, inflection, receptive grammar, expressive syntax
	Phonological memory	Nonword repetition tests such as CN Rep
Verbal short-term memory	Digit span, word span
Decoding specific skills	Letter knowledge	Accuracy of letter-naming, naming letter–sound correspondences
	Phoneme awareness	Phoneme manipulation, phoneme detection, phoneme generation, composite tests where phoneme tasks are in majority
	Rhyme awareness	Rhyme oddity task, rhyme generation tasks, rhyme detection
	Rapid automatized naming (RAN)	Naming speed for symbolic items (objects, letters, digits or colors)
Literacy skills	Word recognition	Word reading accuracy, word reading fluency
	Nonword decoding	Nonword reading accuracy, nonword reading fluency
	Spelling accuracy	Spelling regular words, spelling irregular words, spelling nonwords
	Reading comprehension	Cloze tests or open-ended questions about the meaning of a text

**Table 3 tbl3:** Group Comparisons in Studies of Infants and Toddlers at Family Risk of Dyslexia

Construct	Comparison type	Mean effect size (*d*) [95% CI]	Number of studies [*N* family-risk (*N* control)]	*I*^*2*^	*Tau*^*2*^
General cognitive abilities	Family-risk children vs. controls not at-risk	−.15 [−.61, .31]	1 [49, (28)]	—	—
	Family-risk children with dyslexia vs. controls not at-risk	−.60 [−1.20, .00]*	1 [19, (22)]	—	—
	Family-risk children without dyslexia vs. controls not at-risk	−.05 [−.61, .50]	1 [24, (22)]	—	—
Motor skills	Family-risk children vs. controls not at-risk	.10 [−.14, .34]	2 [137, (124)]	0	0
Articulatory accuracy	Family-risk children vs. controls not at-risk	−.93 [−1.57, −.28]*	1 [19, (20)]	—	—
	Family-risk children with dyslexia vs. controls not at-risk	−1.01 [−1.66,−.37]*	1 [20, (20)]	—	—
	Family-risk children without dyslexia vs. controls not at-risk	−.08 [−.68, .62]	1 [19, (20)]	—	—
Vocabulary knowledge	Family-risk children vs. controls not at-risk	−.32 [−.53, −.12]*	4 [315, (336)]	0	0
	Family-risk children with dyslexia vs. controls not at-risk	−.55 [−1.27, −.24]*	2 [29, (29)]	0	0
	Family-risk children without dyslexia vs. controls not at-risk	−.55 [−1.24, .14]	2 [21, (29)]	0	0
Grammar	Family-risk children vs. controls not at-risk	−1.0 [−1.52, −.47]*	1 [35, (27)]	—	—
*Note*. CI = confidence interval.					
* *p* < 0.05.					

**Table 4 tbl4:** Group Comparisons in Studies of Preschool Children

Construct	Comparison type	Mean effect size (*d*) [95% CI]	Number of studies [*N* family-risk (*N* control)]	*I*^*2*^	*Tau*^*2*^
Panel A: General cognitive abilities and perceptual skills					
Nonverbal IQ	Family-risk children vs. controls not at-risk	−.35 [−.55, −.14]*	7 [315, (336)]	46.40	.04
	Family-risk children with dyslexia vs. controls not at-risk	−.21 [−.48, .06]	6 [201, (276)]	50.59	.06
	Family-risk children without dyslexia vs. controls not at-risk	−.06 [−.30, .18]	6 [227, (242)]	33	.03
Auditory processing	Family-risk children vs. controls not at-risk	−.31 [−.59, −.03]*	2 [100, (100)]	0	0
	Family-risk children with dyslexia vs. controls not at-risk	−.80 [−1.57, −.04]*	1 [9, (22)]	—	—
	Family-risk children without dyslexia vs. controls not at-risk	−.21 [−.77, .35]	1 [26, (22)]	—	—
Visual processing	Family-risk children vs. controls not at-risk	−.11 [−.29, .07]	2 [217, (2148)]	0	0
	Family-risk children with dyslexia vs. controls not at-risk	−.34 [−.82, .13]	1 [26, (47)]	—	—
	Family-risk children without dyslexia vs. controls not at-risk	−.08 [−.59, .43]	1 [21, (47)]	—	—
Motor skills	Family-risk children vs. controls not at-risk	.25 [−.03, .53]	1 [101, (89)]	—	—
Panel B: Oral language skills					
Articulatory accuracy	Family-risk children vs. controls not at-risk	−.79 [−.79, −.01]*	3 [164, (133)]	61.43	.07
	Family-risk children with dyslexia vs. controls not at-risk	−.41 [−.94, .11]	4 [125, (187)]	46.69	.22
	Family-risk children without dyslexia vs. controls not at-risk	−.31 [−.69, .08]	4 [87, (187)]	33.84	.05
Vocabulary knowledge	Family-risk children vs. controls not at-risk	−.65 [−.91, −.38]*	6 [436, (374)]	54.61	.06
	Family-risk children with dyslexia vs. controls not at-risk	−.83 [−1.42, −.24]*	6 [259, (235)]	88.38	.47
	Family-risk children without dyslexia vs. controls not at-risk	−.34 [−.54, −.15]*	5 [236, (192)]	0	0
Grammar	Family-risk children vs. controls not at-risk	−.26 [−.66, .15]	6 [340, (276)]	81.35	.19
	Family-risk children with dyslexia vs. controls not at-risk	−.72 [−1.05, −.40]*	2 [55, (121)]	0	0
	Family-risk children without dyslexia vs. controls not at-risk	−.28 [−.54, −.02]*	3 [101, (134)]	0	0
Phonological memory	Family-risk children vs. controls not at-risk	−.57 [−.79, −.35]*	4 [174, (151)]	0	0
	Family-risk children with dyslexia vs. controls not at-risk	−1.11 [−1.39, −.84]*	5 [98, (180)]	0	0
	Family-risk children without dyslexia vs. controls not at-risk	−.47 [−.72, −.22]*	5 [101, (180)]	0	0
Verbal short-term memory	Family-risk children vs. controls not at-risk	−.45 [−.89, −.04]*	4 [216, (190)]	74.07	.13
	Family-risk children with dyslexia vs. controls not at-risk	−.65 [−1.18, −.12]*	6 [168, (182)]	80.93	.35
	Family-risk children without dyslexia vs. controls not at-risk	−.15 [−.40, .11]	5 [143, (135)]	10.69	.01
Panel C: Decoding specific skills					
Letter knowledge	Family-risk children vs. controls not at-risk	−.47 [−.60, −.34]*	7 [447, (478)]	0	0
	Family-risk children with dyslexia vs. controls not at -risk	−.94 [−1.42, −.45]*	7 [206, (337)]	83.74	.35
	Family-risk children without dyslexia vs. controls not at-risk	−.32 [−.50, −.13]*	7 [226, (290)]	4.41	.002
Phoneme awareness	Family-risk children vs. controls not at-risk	−.56 [−.70, −.43]*	6 [482, (453)]	0	0
	Family-risk children with dyslexia vs. controls not at-risk	−.83 [−1.17, −.48]*	11 [218, (376)]	73.74	.23
	Family-risk children without dyslexia vs. controls not at-risk	−.32 [−.53, −.10]*	10 [262, (350)]	39.28	.05
Rhyme awareness	Family-risk children vs. controls not at-risk	−.90 [−1.36, −.44]*	2 [106, (94)]	54.85	.06
	Family-risk children with dyslexia vs. controls not at-risk	−1.00 [−1.70, −.23]*	1 [37, (25)]	—	—
	Family-risk children without dyslexia vs. controls not at-risk	−.55 [−1.12, .02]	1 [19, (25)]	—	—
Rapid naming	Family-risk children vs. controls not at-risk	−.61 [−.80, −.41]*	6 [381, (385)]	36.08	.02
	Family-risk children with dyslexia vs. controls not at-risk	−1.03 [−1.37, −.69]*	5 [119, (206)]	29.4	.04
	Family-risk children without dyslexia vs. controls not at-risk	−.47 [−.71, −.22]*	5 [185, (206)]	0	0
*Note*. CI = confidence interval.					
* *p* < 0.05.					

**Table 5 tbl5:** Group Comparisons in Studies of Primary School Children

Construct	Comparison type	Mean effect size (*d*) [95% CI]	Number of studies [*N* family-risk (*N* control)]	*I*^*2*^	*Tau*^*2*^
Panel A: General cognitive abilities and perceptual skills					
Nonverbal IQ	Family-risk children vs. controls not at-risk	−.27 [−.50, −.04]*	5 [315, (336)]	35.05	.02
	Family-risk children with dyslexia vs. controls not at-risk	−.19 [−.49, .11]	5 [192, (248)]	55.09	.07
	Family-risk children without dyslexia vs. controls not at-risk	−.02 [−.29, .25]	5 [190, (201)]	38.52	.04
Auditory processing	Family-risk children vs. controls not at-risk	−.48 [−.95, −.02]*	1 [36, (36)]	—	—
Visual processing	Family-risk children vs. controls not at-risk	−.42 [−.75, −.09]*	3 [151, (186)]	43.61	.04
Motor skills	Family-risk children vs. controls not at-risk	.28 [−.01, .57]	1 [98, (85)]	—	—
Panel B: Oral language skills					
Articulatory accuracy	Family-risk children vs. controls not at-risk	−.21 [−1.07, .63]	2 [63, (145)]	80.48	.31
Vocabulary knowledge	Family-risk children vs. controls not at-risk	−.59 [−1.21, .02]	5 [155, (268)]	86.28	.41
	Family-risk children with dyslexia vs. controls not at-risk	−.46 [−.80, −.11]*	3 [74, (63)]	0	0
	Family-risk children without dyslexia vs. controls not at-risk	−.15 [−.46, .18]	3 [105, (63)]	0	0
Grammar	Family-risk children vs. controls not at-risk	−.31 [−.87, .25]	2 [81, (164)]	74.12	.12
Phonological memory	Family-risk children vs. controls not at-risk	−1.02 [−1.42, −.61]*	3 [51, (53)]	0	0
	Family-risk children with dyslexia vs. controls not at-risk	−.83 [−2.07, .41]	2 [70, (102)]	92.18	.74
	Family-risk children without dyslexia vs. controls not at-risk	−.22 [−.65, .22]	2 [86, (102)]	44.53	.04
Verbal short-term memory	Family-risk children vs. controls not at-risk	−.82 [−1.20, −.45]*	4 [100, (88)]	34.66	.05
	Family-risk children with dyslexia vs. controls not at-risk	−1.29 [−1.84, −.73]*	1 [37, (25)]	—	—
	Family-risk children without dyslexia vs. controls not at-risk	−.27 [−.86, .32]	1 [19, (25)]	10.69	.01
Panel C: Decoding specific skills					
Letter knowledge	Family-risk children vs. controls not at-risk	−.41 [−1.20, .41]	3 [47, (93)]	76.15	.62
Phoneme awareness	Family-risk children vs. controls not at-risk	−.69 [−1.09, −.29]*	7 [482, (453)]	70.12	.19
	Family-risk children with dyslexia vs. controls not at-risk	−1.63 [−1.93, −1.33]*	3 [115, (116)]	0	0
	Family-risk children without dyslexia vs. controls not at-risk	−.56 [−.87, −.27]*	3 [158, (116)]	25.08	.02
Rhyme awareness	Family-risk children vs. controls not at-risk	−1.94 [−2.04, −.95]*	1 [37, (29)]	—	—
	Family-risk children with dyslexia vs. controls not at-risk	−1.13 [−1.48, −.79]*	2 [63, (34)]	0	0
	Family-risk children without dyslexia vs. controls not at-risk	−.23 [−.75, .28]	2 [19, (25)]	0	0
Rapid naming	Family-risk children vs. controls not at-risk	−.73 [−1.18, −.28]*	5 [119, (162)]	66.12	.17
	Family-risk children with dyslexia vs. controls not at-risk	−.78 [−1.03, −.53]*	3 [111, (168)]	0	0
	Family-risk children without dyslexia vs. controls not at-risk	−.07 [−.27, .13]	3 [206, (168)]	0	0
Panel D: Literacy skills					
Word recognition	Family-risk children vs. controls not at-risk	−.88 [−1.20, −.66]*	9 [440, (378)]	69.70	.15
	Family-risk children with dyslexia vs. controls not at-risk	−2.44 [−2.80, −2.08]*	9 (15) [246, (354)]	49.70	.14
	Family-risk children without dyslexia vs. controls not at-risk	−.29 [−.47, −.10]*	9 (15) [330, (352)]	0	0
Nonword decoding	Family-risk children vs. controls not at-risk	−1.12 [−1.64, −.60]*	4 [153, (158)]	76.42	.21
	Family-risk children with dyslexia vs. controls not at-risk	−1.83 [−2.23, −1.44]*	6 (10) [163, (264)]	49.59	.11
	Family-risk children without dyslexia vs. controls not at-risk	−.56 [−1.00, −.12]*	6 (10) [257, (264)]	73.99	.21
Spelling accuracy	Family-risk children vs. controls not at-risk	−1.06 [−1.88, −.25]*	3 [37, (29)]	90.83	.45
	Family-risk children with dyslexia vs. controls not at-risk	−2.10 [−2.40, −1.81]*	6 (10) [190, (280)]	9.93	.01
	Family-risk children without dyslexia vs. controls not at-risk	−.42 [−.63, −.21]*	6 (10) [288, (280)]	0	0
Reading comprehension	Family-risk children vs. controls not at-risk	−.77 [−1.14, −.41]*	1 [67, (57)]	—	—
	Family-risk children with dyslexia vs. controls not at-risk	−1.89 [−2.92, −.86]*	3 (4)[64, (110)]	84.50	.91
	Family-risk children without dyslexia vs. controls not at-risk	−.40 [−.91, .12]	3 (4) [50, (110)]	54.40	.15
*Note*. CI = confidence interval.					
* *p* < 0.05.					

**Table 6 tbl6:** Group Comparisons in Studies of Secondary School Children at Family Risk of Dyslexia

Construct	Comparison type	Mean effect size (*d*) [95% CI]	Number of studies [*N* family-risk (*N* control)]
Vocabulary knowledge	Family-risk children with dyslexia vs. controls not at-risk	−1.75 [−2.49, −1.01]*	1 [21, (17)]
	Family-risk children without dyslexia vs. controls not at-risk	−.43 [−1.02, .17]	1 [29, (17)]
Phonological memory	Family-risk children with dyslexia vs. controls not at-risk	−1.16 [−1.83, −.48]*	1 [21, (17)]
	Family-risk children without dyslexia vs. controls not at-risk	−.21 [−.80, .38]	1 [29, (17)]
Phoneme awareness	Family-risk children with dyslexia vs. controls not at-risk	−1.36 [−2.06, −.67]*	1 [21, (17)]
	Family-risk children without dyslexia vs. controls not at-risk	−.26 [−.85, .32]	1 [29, (17)]
Word recognition	Family-risk children with dyslexia vs. controls not at-risk	−1.94 [−2.71, −1.18]*	1 [21, (17)]
	Family-risk children without dyslexia vs. controls not at-risk	−.80 [−1.41, −.19]*	1 [29, (17)]
Spelling accuracy	Family-risk children with dyslexia vs. controls not at-risk	−2.75 [−3.63, −1.87]*	1 [21, (17)]
	Family-risk children without dyslexia vs. controls not at-risk	−.89 [−1.51, −.28]*	1 [29, (17)]
Reading comprehension	Family-risk children vs. controls not at-risk	−.56 [−.99, −.14]*	1 [47, (41)]
	Family-risk children with dyslexia vs. controls not at-risk	−1.00 [−1.67, −.30]*	1 [64, (110)]
	Family-risk children without dyslexia vs. controls not at-risk	−.10 [−.69, .48]	1 [50, (110)]
*Note*. CI = confidence interval.			
* *p* < 0.05.			

**Table 7 tbl7:** Intervention Studies

Construct	Comparison	Mean effect size (*d*) [95% CI]	Number of studies [*N* family-risk (*N* control)]	*I*^*2*^	*Tau*^*2*^
Vocabulary knowledge	At risk controls	0 [−.28, .28]	2 [93, (100)]	0	0
Letter knowledge	At risk controls	.53 [.19, .87]*	5 [232, (194)]	62.41%	.09
	Not at risk controls	.29 [−.31, .89]	3 [135, (122)]	80.70%	.22
Phonological awareness	At risk controls	.55 [.26, .85]*	5 [228, (190)]	57.08	.08
	Not at risk controls	.01 [−.26, .28]	3 [135, (122)]	14.45	.01
Rapid naming	At risk controls	.09 [−.44, .24]	3 [96, (100)]	30.84%	.03
	Not at risk controls	.03 [−.37, .33]	2 [66, (67)]	0	0
Word recognition	At risk controls	.52 [.55, 1.57]*	2 [93, (100)]	90.01	.54
*Note*. CI = confidence interval.					
* *p* < 0.05.					

## Footnotes

1 Scarborough (1989) and Scarborough (1991b) report slightly different prevalence estimates from the same sample. Here the 1991 article is included.

2 A small amount of variance (7%) in reading accuracy in third grade was attributable to parents’ spelling skills.

## Appendix A Characteristics of Studies Comparing Children With Family Risk (Not yet Diagnosed) and Control Children Without Family Risk (Age, Sample Size, Recruitment of Relatives With Dyslexia, Socioeconomic Status, Comparison Type, Constructs Coded, Effect Size for the Difference Between Children With a Family Risk of Dyslexia and Control Children Without Such Risk, and Prevalence of Dyslexia in the Studies Included in the Meta-Analyses)

**Table:**

Study name	Age dyslexia risk (control)	Sample size dyslexia risk (control)	Outcome construct (indicator)	Effect size (*d*)	Recruitment scheme	Socio economic background
Aro et al. 2012 (3)	5 (5)	107 (91)	Vocabulary knowledge (expressive vocabulary BNT)	−.40	Confirmed with testing	No differences in parental education level between the groups
			Vocabulary knowledge (M CDI voc production)			
	2 years 6 months (2 years 6 months)	107 (92)	Grammar (morphological skills)	−.26		
	5 (5)		IQ (WPPSI performance)	−.46		
	8 (8)		Social skills (basic social skills)	−.07		
	5 (5)		Visual skills (visual matching and visual motor precision	.04		
	8 (8)		from NEPSY)	−.30		
			Word reading (reading fluency of text containing 25 sentences)	−.59		
Aro et al. 2009 (3)	8 (8)	101 (89)	IQ (WISC IQ)	−.25	Confirmed with testing	No differences in parental education level between the groups
			Language comprehension	−.44		
			(general language comprehension tests)			
			Maternal education	−.04		
			Motor (m-ABC)	.25		
	3.5 (3.5)		Paternal education	−.09		
			Phon Aw (general phonological skills)	−.64		
			vSTM (general memory skills)	−.39		
Black et al. 2012 (10)	5.52 (5.66)	24 (27)	IQ (WJ brief IQ)	−.87	Parental report of reading disability	No differences in parental educational level
			Maternal education	−.16		
			Maternal skills (verbal IQ WAIS)	−.73		
			Paternal education	−.46		
			Paternal skills (verbal IQ WAIS)	−.37		
			Phon memory CTOPP phon memory (composite vSTM and nonrep)	−.77		
			Vocabulary knowledge (WJ verbal comprehension)	−1.46		
			Visual skills (WJ visual matching)	−.23		
			Word reading (WJ reading mastery test)	−.69		
Boets, Wouters, van Wieringen, & Ghesquière, 2006a (1)	5 (5)	31 (31)	Auditory processing	−.35	Self-report form	Lower education level in the at-risk parents
			(tone in noise detection)			
			Letter knowledge	−.38		
			Phon Aw (composite PA)	−.65		
			Phon memory (nonword repetition)	−.55		
			RAN (color and picture naming)	−.62		
			vSTM (digit span)	.06		
Boets, Wouters, van Wieringen, & Ghesquière, 2006b (1)	5 (5)	31 (31)	Visual processing (visual motion detection)	.19	Self-report form	Lower education level in the at-risk parents
Cardoso-Martins & Pennington, 2004 (6)	5.39 (5.3)	67 (57)	Letter knowledge (letter name)	−.52	—	At-risk group had similar SES
			Nonword reading (WJ word attack)	−.69		
			Phon Aw (Lindamood)	−.26		
			RAN (mean RAN colors, letters, numbers and objects)	−.59		
			Reading comprehension (GORT)	−.78		
			Spelling (WJ spelling)	−.96		
Carroll & Myers, 2010	Reception year or year 1	46 (128)	Articulatory accuracy (DEAP)	.17	Recruited because a parent or sibling had dyslexia	—
			Vocabulary knowledge (expressive vocabulary CELF)	−.02		
			Grammar (sentence structure CELF)	−.05		
Carroll & Snowling, 2004	62.71 (62.53)	17 (17)	Articulatory accuracy (adapted task based on Cookie Monster from *Sesame Street* that mispronounce words)	−.70	Had a parent or sibling with dyslexia	—
			Letter knowledge (knowledge of letters and sounds)	−.02		
			Phon Aw (phoneme matching)	−.37		
			Phon memory (12 two and three syllable nonwords)	−1.30		
			Vocabulary knowledge (BPVS)	−.22		
			Word reading (word recognition early reading)	−.36		
de Bree & Kerkhoff, 2010 (13)	5 years 1 month (5 years 1 month)	57 (26)	Grammar (plural marking)	−.31	Confirmed with testing	No differences in parental educational level
			IQ (Snijders et al IQ test for young children)	−.37		
de Bree et al., 2006 (13)	3 years 3 months (3 years 1 month)	49 (28)	IQ (Snijders et al IQ test for young children)	−.15	Confirmed with testing	No differences in parental educational level
Elbro & Petersen, 2004 (4)	6 years 2 months (6 years 4 months)	47 (41)	Articulatory accuracy (the poor parrot paradigme-pronounciation correction)	−.40	Self-report confirmed with testing	No sing differences in parental education
			IQ (Raven)	−.12		
			Letter knowledge (name 29 letters in Danish)	−.70		
			Maternal education	−.37		
			Nonword reading (nonword reading accuracy)	−.74		
			Paternal education	−1.37		
	6 years 2 months (6 years 4 months)		Phon Aw (phoneme identification)	−.64		
			RAN (30 pictured objects presented three at the time [number correct per minute])	−.47		
	7th grade		Reading comprehension	−.56		
	6 years 2 months (6 years 4 months)		Vocabulary knowledge (PPVT)	−.73		
			vSTM (digit span WISC)	−.37		
	2nd grade		Word reading (word reading accuracy)	−.74		
Eleveld, 2005 (2)	4 years 10 months (4 years 10 months)	117 (52)	Letter knowledge (not specified)	−.41	Self-report confirmed by screening test	The groups were not significantly different at any of the background variables
			Phon Aw (composite phoneme awareness tasks)	−.63		
			RAN (composite colors, objects digits)	−.58		
			Visual skills (visual matching of number letters and figures)	−.19		
	1st grade	67 (25)	Word reading (3 min reading test)	−.28		
Facoetti, Corradi, Ruffino, Gori, & Zorzi, 2010	5.85 (5.67)	20 (67)	Vocabulary knowledge (similarities WISC)	−.07	Assigned based on the adult dyslexia check list	—
			IQ (block design WISC)	.06		
			Letter knowledge (identification of letters)	.05		
			Phon Aw (segmenting syllables by parsing a word into its constituent syllables)	−.05		
			RAN (RAN colors)	−1.17		
			Visual skills (visual cueing task)	−.83		
Gerrits & de Bree, 2009 (13)	3 years 10 months (3 years 6 months)	34 (23)	Articulatory accuracy (percentage of consonants correct)	−.84	Confirmed by testing	—
Gibbs, 2005 (5)	6 years 1 month (6 years 1 month)	37 (29)	Vocabulary knowledge (vocabulary WISC III)	−1.08	Recruited through British dyslexia association	—
			Phon Aw (phoneme deletion)	−1.54		
	3 years 9 months (3 years 9 months)		Vocabulary knowledge (BPVS)	−1.24		
			Rhyme awareness (rhyme awareness)	−1.18		
	6 years 1 month (6 years 1 month)		Rhyme awareness (rhyme oddity)	−1.49		
	3 years 9 months (3 years 9 months)		vSTM (digit span)	−1.16		
	6 years 1 month (6 years 1 month)		vSTM (digit span)	−1.31		
Hindson et al., 2005 (15)	54.6 (55.5)	69 (65)	Auditory processing (speech discrimination)	−.29	Recruited through newspaper ads	More unemployed and single parents in at-risk sample than typical sample
			Grammar (temporal terms)	.51		
			IQ (block design)	−.71		
			Letter knowledge (multiple choice task were the child was asked to point to one out of four letters that was said by the tester)	−.64		
		101 (68)	Phon Aw (phoneme identity task where the child should indicate if two words start at the same sound as a target word)	−.51		
	55 (55.9)	49 (41)	Phon memory (CN Rep)	−.60		
	54.6 (55.5)	69 (65)	Vocabulary knowledge (PPVT)	−.79		
			Rhyme awareness (recognition of rhymes)	−.71		
			Visual skills (visual matching)	−.09		
Hosseini et al., 2013 (10)	5.65 (5.59)	22 (20)	Phon Aw (CTOPP PA)	−.91	Parental report of reading disability	No differences in parental educational level
			RAN (RAN digits)	−.27		
			vSTM (CTOPP phon memory)	−.56		
Kerkhoff, deBree, de Klerk, & Wijnen, 2013 (13)	1 years 6 months (1 years 6 months)	30 (31)	Vocabulary knowledge (M CDI expressive)	−.16	Confirmed with testing	No differences in parental educational level
			Maternal education	−.05		
			Motor (age first walking)	.29		
			Vocabulary knowledge (M CDI receptive)	−.66		
Kevan & Pammer, 2008	67.5 (66.52)	20 (42)	IQ (brief intellectual ability measure Woodcock Johnson III)	−.38	Responded to media campaigns	—
			Letter knowledge (letter word identification WJ III)	.00		
Koster et al., 2005 (2)	17 months	111 (87)	Vocabulary knowledge (number of words produced)	−.30	Volunteers that responded to publicity campaigns	Significantly lower parental education level in the at-risk group
Laakso et al., 1999 (3)	18 months	39 (89)	Vocabulary knowledge (Reynell language production)	−.02	Confirmed with testing	Significantly lower parental education level in the at-risk group
	18 months	111	Vocabulary knowledge (Reynell)	−.02		
Lovio, Naatanen, & Kujala, 2010 (3)	6.7 (6.5)	10 (9)	Letter knowledge (letter knowledge not specified)	−1.55	Confirmed with testing	—
			Phon Aw (NEPSY phonological processing)	−1.28		
			Phon memory (nonrep from NEPSY)	−1.22		
			RAN (RAN colors, numbers and letters)	−1.83		
			vSTM (digit span WISC III)	−.71		
			Word reading (reading syllables and nonwords)	−3.25		
Lyytinen, Ahonen, et al., 2004 (3)	2 years	107 (93)	IQ (Bayley MDI)	−.11	Confirmed with testing	No differences in parental education level between the groups
	5 years	107 (93)	Language comprehension (verbal IQ)	−.44		
	3.5 (3.5)	107 (93)	Sentence rep (NEPSY sentence repetition)	−.13		
Lyytinen, Aro, et al., 2004 (3)	5 (5)	107 (93)	Sentence rep (sentence repetition)	−.17	Confirmed with testing	No differences in parental education level between the groups
Lyytinen, Eklund, & Lyytinen, 2005 (3)	3.5 (3.5)	107 (90)	Vocabulary knowledge (Reynell receptive)	−.20	Confirmed with testing	No differences in parental education level between the groups
	18 months	49 (49)	Vocabulary knowledge (Reynell language skills)	−.02		
Lyytinen, Eklund, Erskine, et al., 2004 (3)	2 years	107 (93)	Motor (Bayley motor index)	.04	Confirmed with testing	No differences in parental education level between the groups
Lyytinen, Poikkeus, et al., 2001 (3)	3.5 (3.5)	106 (94)	Vocabulary knowledge (PPVT)	−.21	Confirmed with testing	No differences in parental education level between the groups
McBride-Chang et al., 2008 (9)	61.06 (61.28)	36 (36)	Auditory processing (tone detection)	−.48	72 children referred to clinical assessment, out of these 36 had a family risk	—
			Grammar (Children were required to combine familiar morphones in new ways to create new structurally legal multi-morphemic nonwords)	−.63		
			Phon Aw (syllable deletion)	−.45		
			RAN (digits)	−.37		
			Word reading (Chinese word recognition)	−.96		
Nash et al., 2013 (14)	3 years 7 months	83 (69)	Articulatory accuracy (PCC)	−.12	Confirmed with testing	Typically developing significantly higher SES than at-risk sample
			Vocabulary knowledge (CELF expressive vocabulary)	−.37		
			Grammar (past tense test)	−.65		
			IQ (two WISC III performance subtests)	−.53		
			Phon memory (PS Rep)	−.67		
			Vocabulary knowledge (CELF basic concepts)	−.50		
Noordenbos, Segers, Seniclaes, Mitterer, & Verhoeven, 2012 (2)	6.93 (6.94)	31 (30)	Nonword reading (3 min nonword reading test)	−1.28	Confirmed with testing	—
			Phon Aw (phoneme segmentation and phoneme deletion)	−.49		
			RAN (RAN letters)	−.52		
			vSTM (repeating words and sentences)	−.62		
			Word reading (3 min reading test)	−1.20		
Puolakanaho, Poikkeus, Ahonen, Tolvanen, & Lyytinen, 2004 (3)	3 years 5 months	98 (91)	Phon Aw (PA mean; word, syllable level, phonological units)	−.52	Confirmed with testing	—
Raschle, Chang, & Gaab, 2011 (11)	5 years 11 months (5 years 7 months)	10 (10)	Vocabulary knowledge (CELF vocabulary knowledge)	−.70	Confirmed with testing	No significant SES difference
			Grammar (CELF language structure)	−.31		
			Parental education	−.05		
Raschle, Stering, Meissner, & Gaab, 2013 (11)	5 years 9 months (5 years 6 months)	14 (14)	Grammar (VAT inflection)	−.37	Confirmed with testing	No significant SES difference
			IQ (K-BIT)	−.10		
			Phon Aw (deletion)	−.63		
			Phon memory (nonrep)	−.05		
			RAN (objects)	−1.75		
			Vocabulary knowledge (CELF)	−.28		
Richardson, Kulju, et al., 2009 (3)	24.00 (24)	19 (20)	Articulatory accuracy (amount of unintelligible speech)	.93	—	—
	30.00 (30)	19 (20)	Vocabulary knowledge (mean length of utterance)	−.41		
Torkildsen, Syversen, et al., 2007	24 (24)	9 (17)	Vocabulary knowledge (M CDI expressive)	−.93	Recruited through newspaper ads, diagnosed by municipal authorities in school	—
Torppa, Georgiou, et al., 2012 (3)	6 years 5 months	105 (90)	Phon Aw (composite)	−.40	Confirmed with testing	No significant difference in education levels
			RAN (composite objects, colors, numbers)	−.50		
	7 (7)	105 (90)	Spelling (spelling nonwords)	−.31		
			Word reading (text reading accuracy)	−.39		
Torppa et al., 2007 (3)	5.5 (5.5)	96 (90)	Letter knowledge (letter knowledge)	−.47	Confirmed with testing	—
			Phon Aw (phonological awareness composite)	−.48		
			Vocabulary knowledge (receptive vocabulary)	−.36		
van Alphen, de Bree, et al., 2004 (13)	319.17 months	35 (27)	Grammar (plurals)	−1.00	Confirmed with testing	—
Vandermosten, Boets, et al., 2011 (1)	11.6 (11.7)	13 (25)	IQ (Raven)	−.32	—	—
			Nonword reading	−2.16		
			Spelling	−2.19		
			Word reading	−2.33		
Viholainen, Ahonen, et al., 2006 (3)	3 years 6 months (3 years 6 months)	13 (25)	Vocabulary knowledge (Boston naming test)	−.43	Confirmed with testing	—
	3 years 6 months (3 years 6 months)	75 (79)	Vocabulary knowledge (PPVT)	−.20		
Viholainen, Aro, et al., 2011 (3)	8 years 6 months	98 (85)	IQ (WISC full scale)	−.20	Confirmed with testing	—
	6 years 6 months	98 (85)	Motor (m-ABC)	.28		
	9 (9)	98 (85)	Word reading (word list reading)	−.52		
*Note.* BAS = British Ability Scales; Bayley MDI = Bayley Mental Developmental Index; BNT = Boston Naming Test; BPVS = British Picture Vocabulary Scale; CELF = Clinical Evaluation of Language Fundamentals; CN Rep = Children’s Test of Nonword Repetition; Comp = comprehension; DAS = Differential Ability Scales; CPM = Colored Progressive Matrices; DEAP = Diagnostic Evaluation of Articulation and Phonology; GORT = Gray Oral Reading Tests; K-BIT = Kaufman Brief Intelligence Test; m-ABC = Movement Assessment Battery for Children; M CDI = MacArthur-Bates Communicative Development Inventories; NARA = Neale Analysis of Reading Ability; NEPSY = Developmental Neuropsychological Assessment; PA = phonological awareness; PCC = percentage consonants correct; Phon Aw = phonological awareness; Phon memory = phonological memory; PPVT = Peabody Picture Vocabulary Test; RAN = rapid naming; SON-R = Snijders-Oomen nonverbal intelligence tests; vSTM = verbal short-term memory; WAIS = Wechsler Adult Intelligence Scale; WPPSI = Wechsler Preschool and Primary Scale of Intelligence; WISC = Wechsler Intelligence Scale for Children; WJ = Woodcock Johnson; WOLD = Wechsler Objective Language Dimensions; WORD = Wechsler Objective Reading Dimensions; Word ID = word identification; WRMT= Woodcock Reading Mastery Tests; YARC = York Assessment of Reading for Comprehension.						

## Appendix B Characteristics of Studies Comparing Children With Family Risk Diagnosed With Dyslexia and Control Children Without Family Risk (Age, Sample Size, Recruitment of Relatives With Dyslexia, Socioeconomic Status, Comparison Type, Constructs Coded, Effect Size for the Difference Between Children With a Family Risk of Dyslexia and Control Children Without Such Risk, and Prevalence of Dyslexia in the Studies Included in the Meta-Analyses)

**Table:**

Study name	Age when effect size was coded family-risk (control)	Sample size Group 1 (Group 2)	Construct (outcome test)	Effect size (*d*)	Prevalence of dyslexia (in family-risk sample/in control sample without family risk)	Recruitment of relatives with dyslexia	Socioeconomic status
Blomert & Willems, 2010	Kgarten	53 (47)	Dyslexia versus controls:		44%/.09%	Parental questionnaire, sibling with a diagnosis of dyslexia	—
			Articulatory accuracy (pronounce a word correctly after parts have been deleted)	.09			
			IQ (general intelligence test not specified) Verbal short term memory (word span)				
			Receptive vocabulary (test not specified)	.19			
			Letter knowledge (correctly name or sound out visual presented letters)	.18			
			Phon Aw (blend phonemes or phoneme strings)	.37			
				.11			
				.10			
Boets, De Smedt, et al., 2010 (1)			Dyslexia versus controls:			—	Maternal and paternal education are measured
	5.33 (5.33)	16 (26)	IQ (Raven)	−.32			
			Phon Aw (composite PA)	−1.37			
			Phon memory (CN Rep)	−1.23			
			RAN (colors and objects)	−.80			
			vSTM (digit span)	−.40			
	1st grade		Vocabulary knowledge (WISC vocabulary)	−.49			
			Nonword reading (nonword reading fluency)	−1.56			
			Paternal education	.31			
			Maternal education	.00			
			Unaffected family-risk versus controls:		44%	—	Maternal and paternal education are measured
	5.33 (5.33)	20 (26)	IQ (Raven)	−.32			
			Phon Aw (composite PA)	−.52			
			Phon memory (CN Rep)	−.65			
			RAN (colors and objects)	−.32			
			vSTM (digit span)	.25			
	1st grade		Vocabulary knowledge (WISC vocabulary)	−.40			
			Nonword reading (nonword reading fluency)	−.27			
			Paternal education	−.28			
			Maternal education	.00			
Boets, Vandermosten, et al., 2011 (1)	5:6 (5:6)	16 (26)	Dyslexia versus controls:		44%	—	Groups matched on parental education level
			IQ (WISC III block design)	.00			
			Vocabulary knowledge (WISC III vocabulary)	−.40			
			Spelling (spelling test Dudal, 1997)	−1.92			
	6:10 (6:10)		Word reading (word reading test)	−2.53			
		20 (26)	Unaffected family-risk versus controls:				Matched
	5:6 (5:6)		IQ (WISC III block design)	.00			
			Language comprehension (WISC III vocabulary)	.00			
	6:10 (6:10)		Spelling (spelling test Dudal, 1997)	−.59			
			Word reading (word reading test)	−.43			
Boets, Wouters, van Wieringen, et al., 2007 (1)			Dyslexia versus controls:		29%/.09%	At least one first-degree relative diagnosed with dyslexia	Significant differences in maternal or paternal level between the at-risk group and the control group
	63 (64)	9 (28)	Rhyme awareness (rhyme identity)	−1.00			
			Auditory processing (speech perception)	−.80			
			IQ (Raven CPM)	−.23			
			Phon Aw (composite phoneme and rhyme identity tasks)	−1.29			
			Phon memory (Dutch adaption of CN Rep)	−.85			
			RAN (rapid naming of colors and objects)	−.95			
	64 (64)	22 (28)	Unaffected family-risk versus controls:		—	At least one first-degree relative diagnosed with dyslexia	
			Rhyme awareness (rhyme identity)	−.21			
			Auditory processing (speech perception)	−.55			
			IQ (Raven CPM)	−.33			
			Phon Aw (composite phoneme and rhyme identity tasks)	−.65			
			Phon memory (Dutch adaption of CN Rep)	−.67			
			RAN (rapid naming of colors and objects)	−.50			
Carroll, Mundy, & Cunningham, 2014	67.67 (64.12)	78 (17)	Dyslexia versus controls:		44.7%/17%	At least one first-degree relative diagnosed with dyslexia after a professional assessment	
			Articulatory accuracy (judging pronunciation of words)	−1.09			
			Phon Aw (phoneme matching)	−.83			
			Phon memory (CN Rep)	−1.27			
			Vocabulary knowledge (CELF)	−1.34			
			Letter knowledge	−.51			
			Word reading (YARC)	−2.81			
			Reading comprehension (YARC)	−1.11			
		78 (21)	Unaffected family-risk versus controls:				
			Articulatory accuracy (judging pronunciation of words)	−.34			
			Phon Aw (phoneme matching)	−.11			
			Phon memory (CN Rep)	−.07			
			Vocabulary knowledge (CELF)	−.37			
			Letter knowledge	−.00			
			Word reading (YARC)	−.51			
			Reading comprehension (YARC)	−.42			
de Bree, Wijnen, & Gerrits, 2010 (13)			Dyslexia versus controls:	−1.16	50%/22%	Standardized tests of parents	—
	4:4 (4:5)	19 (23)	Phon memory (Dutch version of Dollaghan & Campbell, 1998)				
			Unaffected family-risk versus controls:		—	Standardized tests of parents	—
	4:4 (4:5)	19 (23)	Phon memory (Dutch version of Dollaghan & Campbell, 1998)	−.66			
Elbro & Petersen, 2004 (4)	7th grade	47 (41)	Only prevalence included in the meta-analysis since test data were reported for at-risk children versus controls	—	55%/20%	Self-report and test	Fathers in the not-at-risk group had significantly higher education than fathers in the at-risk group
Elbro, Borstrøm, & Petersen, 1998 (4)			Dyslexia versus controls:		37%/11%	Self-report and test	Fathers in the not-at-risk group had significantly higher education than fathers in the at-risk group
	2nd grade	18 (42)	Articulatory accuracy (articulatory skills) grammar (inflection formation) IQ (Raven) letter knowledge	−.59			
			Phon Aw (phoneme discrimination) RAN (picture naming) vocabulary knowledge (language PPVT)	−.59			
			vSTM (WISC-R)	−.26			
				−1.17			
				−.89			
				−.48			
				−.90			
				−.81			
			Unaffected family-risk versus controls:			Self-report and test	Fathers in the not-at-risk group had significantly higher education than fathers in the at-risk group
	2nd grade	31 (42)	Articulatory accuracy (articulatory skills)	−.59			
			Grammar (inflection formation)	−.27			
			IQ (Raven)	−.26			
			Letter knowledge	−.63			
			Phon Aw (phoneme discrimination)	−.47			
			RAN (picture naming)	−.51			
			Vocabulary knowledge (language PPVT)	−.37			
			vSTM (WISC-R)	−.20			
Gallagher, Frith, & Snowling, 2000 (5)			Dyslexia versus controls:		66%/16%	Confirmed with standardized testing	Middle-class volunteer sample
	6 years (6 years)	63 (34)	Rhyme awareness (rhyme knowledge) vSTM (digit span forward)	−1.00			
	45 months			−1.35			
			Unaffected family-risk versus controls:		66%/16%	Confirmed with standardized testing	Middle-class volunteer sample
	6 years (6 years)		Rhyme awareness (rhyme knowledge) vSTM (digit span forward)	−.22			
	45 months			−.35			
Ho, Leung, & Cheung, 2011		36 (25)	Dyslexia versus controls:		47%	Confirmed with standardized testing	At-risk parents had significantly lower education and income than the controls
	5:9 (6:18)		IQ (Raven CPM)	.55			
			Vocabulary knowledge (adapted word definitions)	−.67			
			Vocabulary knowledge (picture vocabulary)	−1.17			
			Phon Aw (syllable deletion)	−1.36			
			RAN (rapid naming of colors and objects)	−.58			
			Spelling (Chinese spelling)	−2.40			
			Word reading (Chinese word reading)	−1.82			
		40 (25)	Unaffected family-risk versus controls:			Confirmed with standardized testing	At-risk parents had significantly lower education and income than the controls
	6:6 (6:18)		IQ (Raven CPM)	−.17			
			Vocabulary knowledge (adapted word definitions)	−.14			
			Vocabulary knowledge (picture vocabulary)	−.29			
			Phon Aw (syllable deletion)	−.23			
			RAN (rapid naming of colors and objects)	−.05			
			Spelling (Chinese spelling)	−.11			
			Word reading (Chinese word reading)	.15			
Leppänen, Hämäläinen, et al., 2010 (3)			Dyslexia versus controls:		—	Confirmed with standardized testing	—
	3.5 (3.5)	8 (25)	Phon Aw (composite PA)	−.85			
	5.0 (5.0)		vSTM (composite vSTM)	−.86			
	5.5 (5.5)		RAN (composite RAN)	−1.61			
	9.0 (9.0)		Word reading (composite word reading accuracy and fluency)	−2.78			
			Spelling (composite)	−1.94			
			Unaffected family-risk versus controls:		—	Confirmed with standardized testing	
	3.5 (3.5)	14 (25)	Phon Aw (composite)	−.83			
	5.0 (5.0)		vSTM (composite)	−.39			
	5.5 (5.5)		RAN (composite)	−.15			
	9.0 (9.0)		Spelling (composite)	−.02			
			Word reading (composite word reading accuracy and fluency)	−.05			
McBride-Change et al., 2011 (9)			Dyslexia versus controls:			Merged at-risk sample, both family-risk and delayed language development	—
	Kgarten	26 (47)	IQ (not specified)	.23			
			Phon Aw (syllable deletion)	−.54			
			RAN (digits)	−1.13			
			Visual skills (Gardener test of visual perception)	−.34			
	7:3		Word reading (Chinese character reading)	−2.50			
			Unaffected family-risk versus controls:			Merged at-risk sample, both family-risk and delayed language development	—
	Kgarten	21 (47)	IQ (not specified)	.03			
			Phon Aw (syllable deletion)	−.41			
			RAN (digits)	−.56			
			Visual skills (Gardener test of visual perception)	−.08			
	7:3		Word reading (Chinese character reading)	−.33			
Pennala, Eklund, et al., 2010 (3)	3rd grade	33 (77)	Dyslexia versus controls:				—
			Phon memory (NEPSY nonrep)	−.21		Confirmed with standardized testing	
			RAN (naming speed composite)	−.70			
			Spelling (word spelling)	−1.85			
			Word reading (word reading accuracy)	−2.39			
	3rd grade	68 (80)	Unaffected family-risk versus controls:			Confirmed with standardized testing	—
			Phon memory (nonrep NEPSY)	−.05			
			RAN (nonrep composite)	−.00			
			Spelling (word spelling)	−.37			
			Word reading (word reading accuracy)	−.28			
Pennington & Lefly, 2001 (6)			Dyslexia versus controls		34%/.06%	Reading history questionnaire that in cases of doubt were supplemented by testing	Matched on SES
	5:4 (5:3)	22 (54)	Letter knowledge (letter name knowledge)	−1.32			
	5:4 (5:3)	42 (54)	Unaffected family-risk versus controls:			Reading history questionnaire that in cases of doubt were supplemented by testing	Matched on SES
			Letter knowledge (letter names)	−.08			
	End of second grade	22 (54)	Dyslexia versus controls:			Reading history questionnaire that in cases of doubt were supplemented by testing	Matched on SES
			IQ (WISC vocabulary and block design subtests)	−.31			
			Nonword reading (WJ word attack)	−1.29			
			Spelling (WRAT spelling)	−1.89			
			Word reading (WJ letter word identification)	−1.75			
	End of second grade	42 (54)	Unaffected family-risk versus controls:			Reading history questionnaire that in cases of doubt were supplemented by testing	Matched on SES
			IQ (WISC vocabulary and block design subtests)	−.22			
			Nonword reading (WJ word attack)	−.39			
			Spelling (WRAT spelling)	−.69			
			Word reading (WJ letter word identification)	−.50			
Plakas, van Zuijen, et al., 2013 (2)	3:4 (3:4)	10 (13)	Dyslexia versus controls:			Confirmed with standardized testing	—
			IQ (SON-R)	−.60			
			Language comprehension (Reynell)	−.74			
			Phon Aw (deletion)	−2.37			
			vSTM (word span)	−.80			
	3:4 (3:4)	15 (13)	Unaffected family-risk versus controls:			Confirmed with standardized testing	—
			IQ (SON-R)	−.22			
			Language comprehension (Reynell)	.26			
			Phon Aw (deletion)	−.52			
			vSTM (word span)	.26			
Plakas, van Zuijen, et al., 2013 (2)	41 months	15 (13)	Unaffected family-risk versus controls:			Confirmed with standardized testing	—
			Grammar (expressive syntax)	.17			
Regtvoort, van Leeuwen, et al., 2006 (2)	5:6 (5)	11 (12)	Dyslexia versus controls:			Confirmed with standardized testing	—
			Nonword reading (1-min nonword reading test)	−.99			
			Spelling (spelling test)	−1.84			
			Word reading (1-min reading test)	−2.72			
	5:6 (5)	15 (12)	Unaffected family-risk versus controls:			Confirmed with standardized testing	—
			Nonword reading (1-min nonword reading test)	−.72			
			Spelling (spelling test)	−.29			
			Word reading (1 min reading test)	−.16			
Scarborough, 1990 (7)			Dyslexia versus controls:			Confirmed with standardized testing	Matched on SES
	30 months	20 (20)	Articulatory accuracy (consonant errors)	−1.01			
			Vocabulary knowledge (BNT)	−.50			
			Vocabulary knowledge (PPVT)	−.86			
	2nd grade		IQ (WISC)	−.42			
			Word reading (WJ reading cluster)	−2.99			
			Unaffected family-risk versus controls:			Confirmed with standardized testing	Matched on SES
	30 months	12 (20)	Articulatory accuracy (consonant errors)	−.08			
			Vocabulary knowledge (PPVT)	−.20			
			Vocabulary knowledge (BNT)	−.32			
	2nd grade		IQ (WISC)	.28			
			Word reading (WJ reading cluster)	−.03			
Scarborough, 1991a (7)	2nd grade	35 (44)	Only prevalence coded since means and *SD*s on single tests were not reported.		65%/.05%	Confirmed with standardized testing	Matched on SES
Smith, 2009 (8)	8:9 (8:9)	10 (7)	Dyslexia versus controls:		58%	Confirmed with standardized testing	Mothers in high-risk sample had significantly lower education level than mothers in control sample
			Maternal education	−1.20			
			Nonword reading (WJ word attack)	−.78			
			Word reading (WRMT-R word ID)	−2.56			
			Reading comprehension	−2.30			
	8:9 (8:9)	10 (7)	Unaffected family-risk versus controls:	−1.83		Confirmed with standardized testing	Mothers in high-risk sample had significantly lower education level than mothers in control sample
			Maternal education	.75			
			Nonword reading (WJ word attack)	.30			
			Word reading (WRMT-R word ID)	.36			
			Reading comprehension				
Smith, Roberts, Locke, & Tozer, 2010 (8)	8–19 months	6 (5)	Unaffected family-risk versus controls:			Confirmed with standardized testing	Mothers in high-risk sample had significantly lower education level
			Articulatory accuracy (syllable index score)	−.71			
			Language comprehension (general conceptual ability)	−.88			
Smith, Smith, et al., 2008 (8)			Dyslexia versus controls:			Confirmed with standardized testing	Mothers in high-risk sample had significantly lower education level
	Age 2–3	9 (9)	Vocabulary knowledge (DAS expressive vocabulary)	−.71			
			IQ (Bayley comprehension)	−.60			
			Vocabulary knowledge (DAS language comprehension)	−.95			
			vSTM (DAS digit recall)	−.79			
			Nonword reading (WRMT word attack)	−2.96			
			Reading comprehension (GORT	−1.17			
	Grade school		Word reading (WRMT word ID)	−5.46			
			Unaffected family-risk versus controls:			Confirmed with standardized testing	Mothers in high-risk sample had significantly lower education level
	Age 2–3	9 (9)	Vocabulary knowledge (DAS expressive vocabulary)	−.88			
			IQ (Bayley comprehension)	.19			
			Vocabulary knowledge (DAS language comprehension)	−1.08			
			vSTM (DAS digit recall)	−.58			
	Grade school		Nonword reading (WRMT word attack)	−.42			
			Reading comprehension (GORT	−.19			
			Word reading (WRMT word ID)	−.69			
Snowling et al., 2003 (5)	3:9 (3:9)	37 (25)	Dyslexia versus controls:		66%/16%	Confirmed with standardized testing	Middle-class volunteer sample
			Articulatory accuracy (Edinburgh articulation test)	−.14			
			Rhyme awareness (nursery rhymes)	−1.20			
			IQ (Draw A Man [Harris, 1963])	−.45			
			Vocabulary knowledge (BPVS)	−1.40			
			Letter knowledge (identify 26 letters by name and sound)	−1.06			
			Phon Aw (nursery rhyme knowledge)	−1.21			
			Phon memory (CN Rep)	−.99			
	3:9 (3:9)	19 (25)	Unaffected family-risk versus controls:			Confirmed with standardized testing	Middle-class volunteer sample
			Articulatory accuracy (Edinburgh articulation test)	.20			
			Rhyme awareness (nursery rhymes)	−.54			
			IQ (Draw a man (Harris, 1963)	.62			
			Language comprehension (BPVS)	−.40			
			Vocabulary knowledge (BPVS)	−.40			
			Letter knowledge (identify 26 letters by name and sound)	−.48			
			Phon Aw (nursery rhyme knowledge)	−.54			
			Phon memory (CN Rep)	−.53			
	8 years	37 (25)	Dyslexia versus controls:			Confirmed with standardized testing	Middle-class volunteer sample
			IQ (composite BAS Matrices and BAS Recall of Designs)	−.65			
			Vocabulary knowledge (WOLD listening comprehension)	−1.66			
			Nonword reading (Graded Nonword Reading Test [Snowling et al. 1996])	−2.36			
			Phon Aw (Phoneme deletion)	−1.58			
			Phon memory (nonword repetition three and four syllable)	−1.48			
			Reading comprehension (WORD reading comprehension)	−3.04			
			Spelling (WORD spelling)	−2.80			
			Word reading (WORD reading)	−2.79			
			Rhyme awareness (rhyme oddity)	−1.30			
	8 years	19 (25)	Unaffected family-risk versus controls:			Confirmed with standardized testing	Middle-class volunteer sample
			IQ (composite BAS Matrices and BAS Recall of Designs)	−.29			
			Vocabulary knowledge (WOLD listening comprehension)	−.47			
			Nonword reading (Graded Nonword Reading Test (Snowling et al. 1996))	−1.29			
			Phon Aw (phoneme deletion)	−.80			
			Phon memory (nonword repetition three and four syllable)	−.52			
			Reading comprehension (WORD reading comprehension)	−1.02			
			Spelling (WORD spelling)	−.25			
			Word reading (WORD reading)	−.60			
			Rhyme awareness (rhyme oddity)	−.24			
Snowling, Muter, & Carroll, 2007 (5)	12–13	21 (17)	Dyslexia versus controls:			Confirmed with standardized testing	Middle-class volunteer sample
			Vocabulary knowledge (WASI vocabulary)	−1.75			
			Nonword reading (from Griffiths & Snowling, 2001)	−1.82			
			Phon Aw (Phoneme deletion)	−1.36			
			Phon memory (CN Rep)	−1.16			
			Reading comprehension (NARA)	−1.05			
			Spelling (WORD spelling)	−2.75			
			Word reading (exception word reading)	−1.93			
	12–13	29 (17)	Unaffected family-risk versus controls:	−.43		Confirmed with standardized testing	Middle-class volunteer sample
			Vocabulary knowledge (WASI vocabulary) Nonword reading (from Griffiths & Snowling, 2001)	−.37			
			Phon Aw (phoneme deletion)	−.26			
			Phon memory (CN Rep)	−.21			
			Reading comprehension (NARA)	−.10			
			Spelling (WORD spelling)	−.89			
			Word reading (exception word reading)	−.80			
Torppa, Lyytinen, Erskine, et al., 2010 (3)	5 years	37 (84)	Dyslexia versus controls:			Confirmed with testing	Mothers of the children in the dyslexia affected group had on average a lower education level than children in the two other groups
			Vocabulary knowledge (PPVT)	−.65			
			Letter knowledge (nonstandardized task)	−1.35			
			Phon Aw (phonological sensitivity [NEPSY])	−.76			
			RAN (Denckla & Rudel, 1976)	−1.06			
			Grammar (inflectional morphology adverb and past tense)	−.79			
	5 years	68 (84)	Unaffected family-risk versus controls:				
			Vocabulary knowledge (PPVT)	−.32			
			Letter knowledge (nonstandardized task)	−.33			
			Phon Aw (phonological sensitivity [NEPSY])	−.28			
			RAN (Denckla & Rudel, 1976)	−.25			
			Grammar (inflectional morphology adverb and past tense)	−.37			
	Second grade	37 (84)	Dyslexia versus controls:		35%/10%	Confirmed with testing	Mothers of the children in the dyslexia affected group had on average a lower education level than children in the two other groups
			Nonword reading (nonword reading)	−1.65			
			Spelling (spelling words and nonwords)	−1.71			
			Word reading (word reading accuracy in meaningful text)	−1.38			
	Second grade	68 (84)	Unaffected family-risk versus controls:			Confirmed with testing	Mothers of the children in the dyslexia affected group had on average a lower education level than children in the two other groups
			Nonword reading (nonword reading)	−.17			
			Spelling (spelling words and nonwords)	−.16			
			Word reading (word reading accuracy in meaningful text)	−.04			
van Bergen, de Jong, Plakas, et al., 2012 (2)	48 (48)	42 (66)	Dyslexia versus controls:		30%/.03%	Confirmed with testing	Children in at-risk group had parents (both mothers and fathers) with significantly lower education than in the control sample
			IQ (SON-R nonverbal IQ test)	−.53			
			Unaffected family-risk versus controls:				
			IQ (SON-R nonverbal IQ test)	−.03			
	98 (96)	42 (66)	Dyslexia versus controls:			Confirmed with testing	Children in at-risk group had parents (both mothers and fathers) with significantly lower education than in the control sample
			Nonword reading (nonword reading accuracy [de Jong & Wolters, 2002])	−2.06			
			Phon Aw (phoneme deletion)	−1.84			
			RAN (digits and colors)	−.98			
			Spelling (write down words presented in a sentence)	−1.74			
			Word reading (word reading accuracy test)	−2.42			
	98 (96)	99 (66)	Unaffected family-risk versus controls:			Confirmed with testing	Children in at-risk group had parents (both mothers and fathers) with significantly lower education than in the control sample
			Nonword reading (nonword reading accuracy [de Jong & Wolters, 2002])	−.56			
			Phon Aw (phoneme deletion)	−.66			
			RAN (digits and colors)	−.15			
			Spelling (write down words presented in a sentence)	−.37			
			Word reading (word reading accuracy test)	−.64			
van Bergen, de Jong, Regtvoort et al. 2011 (2)	70.4 (70)	22 (12)	Dyslexia versus controls:		32%	Confirmed with testing	Children in at-risk group had parents (both mothers and fathers) with significantly lower education than in the control sample
			Letter knowledge (receptive letter knowledge task)	−1.42			
			Phon Aw (phoneme blending and segmentation)	−.10			
			RAN (colors and object)	−1.39			
			Word reading (3 min reading test)	−3.13			
	70.6 (70)	45 (12)	Unaffected family-risk versus controls:			Confirmed with testing	Children in at-risk group had parents (both mothers and fathers) with significantly lower education than in the control sample
			Letter knowledge (receptive letter knowledge task)	−.54			
			Phon Aw (phoneme blending and segmentation)	.02			
			RAN (colors and object)	−.64			
			Word reading (3 min reading test)	−2.16			
			Dyslexia versus controls:				
	Grade 1	22 (12)	IQ (Raven)	−.37			
			Language comprehension (similar to PPVT)	−.11			
	Grade 5	22 (12)	Nonword reading (2 min pseudoword test)	−4.60			
	Grade 1	45 (12)	Unaffected family-risk versus controls:				
			IQ (Raven)	−.22			
	Grade 5	45 (12)	Language comprehension (similar to PPVT)	−.09			
			Nonword reading (2 min pseudoword test)	−2.92			
*Note.* BAS = British Ability Scales; Bayley MDI = Bayley Mental Developmental Index; BNT = Boston Naming Test; BPVS = British Picture Vocabulary Scale; CELF = Clinical Evaluation of Language Fundamentals; CN Rep = Children’s Test of Nonword Repetition; Comp = comprehension; DAS = Differential Ability Scales; CPM = Colored Progressive Matrices; DEAP = Diagnostic Evaluation of Articulation and Phonology; GORT = Gray Oral Reading Tests; K-BIT = Kaufman Brief Intelligence Test; m-ABC = Movement Assessment Battery for Children; M CDI = MacArthur-Bates Communicative Development Inventories; NARA = Neale Analysis of Reading Ability; NEPSY = Developmental Neuropsychological Assessment; PA = phonological awareness; PCC = percentage consonants correct; Phon Aw = phonological awareness; Phon memory = phonological memory; PPVT = Peabody Picture Vocabulary Test; RAN = rapid naming; SON-R = Snijders-Oomen nonverbal intelligence tests; vSTM = verbal short-term memory; WAIS = Wechsler Adult Intelligence Scale; WPPSI = Wechsler Preschool and Primary Scale of Intelligence; WISC = Wechsler Intelligence Scale for Children; WJ = Woodcock Johnson; WOLD = Wechsler Objective Language Dimensions; WORD = Wechsler Objective Reading Dimensions; Word ID = word identification; WRMT = Woodcock Reading Mastery Tests; YARC = York Assessment of Reading for Comprehension.							

## Appendix C Characteristics of Intervention Studies Concerning Children with Family Risk of Dyslexia

**Table:**

Study name	Comparison	Time point	Sample size *T* (*C*)	Outcome	Effect size (*d*)	Age *T* (*C*)	Design	Intervention type/Intervention duration
Duff et al., (2015) (14)	Trained at risk vs. controls at risk.	Posttest	67 (77)	Language comprehension	.03	6 years (6 years)	Randomized waiting list design	Reading and language intervention (RALI), phoneme awareness/decoding, and language comprehension skills
				Letter knowledge				One group 9 weeks of intervention, the other group 18 weeks of intervention alternating between 20 and 30 min group and individual daily sessions. Implemented by teaching assistants.
				Nonword reading	.24			
				Phon Aw	.17			
				Spelling	.40			
				Word reading	−.21			
					.00			
Elbro & Petersen, 2004 (4)	Trained at risk vs. controls at risk	Posttest	35 (47)	Letter knowledge	.99	6:2(6:2)	Nonrandomized pre/posttest control group design	Training program written for the study, inspired by the Lundberg and the Lindamood programs. Focused on phoneme-awareness training and letter knowledge. Intervention was 30 min a day for 17 weeks, delivered by kindergarten teachers.
				Phon Aw	1.14			
				RAN	−.22			
	Trained at risk vs. controls not at risk	Posttest	35 (41)	Letter knowledge	.39			
				Phon Aw	.24			
				RAN	.06			
	Trained at risk vs. controls at risk	Follow-up gain 2nd–3rd grade	35 (47)	Nonword reading	−.01			
				Word reading	−.18			
	Trained at risk vs. controls not at risk	Follow-up gain 2nd–3rd grade	35 (41)	Nonword reading	−.04			
				Word reading	−.17			
Fielding-Barnsley, & Purdie, 2003	Trained at risk vs. controls at risk	Posttest	26 (23)	Language comprehension	−.08	70.2 (70.5)	Nonrandomized pre/posttest control group design	Dialogic reading intervention where parents were asked to read to their children using the principles of dialogic reading. Five times during the 8-week intervention.
				Phon Aw				
				Spelling	.23			
				Word reading	1.26	1st grade		
					1.09			
Hindson et al., 2005 (15)	Trained at risk vs. controls at risk	Posttest	69 (17)	Letter knowledge	.12	54.6 (55.5)	Nonrandomized pre/posttest control group design	The sound foundations package based on training the children on a limited set of phonemes. The intervention was delivered by one of two teachers from research group either at home or in school. Each session 30 min, 11–17 sessions within a period of 3 months
				Ph awareness	.88			
	Trained at risk vs. controls not at risk	Posttest	69 (65)	Letter knowledge	−.22			
				Phon Aw	−.18			
Regtvoort & van der Leij, 2007 (2)	Trained at risk vs. controls at risk	Posttest	31 (26)	Letter knowledge	.87	5:7 (5:7)	Nonrandomized pre/posttest control group design	Word building (Beck 1989). Focuses on reading instruction, letter–sound correspondence, and phonemic awareness. Children were trained by their parents, 10 min a day, 5 days a week for 14 weeks.
				Phon Aw	.48			
				RAN	−.32			
	Trained at risk vs. controls not at risk	Posttest	31 (16)	Letter knowledge	.84			
				Phon Aw	.13			
				RAN	−.19			
	Trained at risk vs. controls at risk	Follow-up gain middle 1st grade to end 1st	31 (26)	RAN	−.09			
				Word reading	−.16			
	Trained at risk vs. controls not at risk	Follow-up gain middle 1st grade to end 1st	31 (16)	RAN	−.14			
				Word reading	−.15			
	Trained at risk vs. controls at risk	Follow-up gain end of 1st grade to end of 2nd	31 (26)	Nonword reading	.46			
				RAN	.20			
				Spelling	−.17			
				Word reading	−.10			
	Trained at risk vs. controls not at risk	Follow-up gain end of 1st grade to end of 2nd	31 (16)	Nonword reading	.26			
				RAN	.75			
				Spelling	−.58			
				Word reading	.01			
van Otterloo & van der Leij, 2009 (2)	Trained at risk vs. controls at risk	Posttest	30 (27)	Letter knowledge	.50	69.87 (71.13)	Posttest-only control group design	Sounding sounds and Jolly letters–a home-based prereading program with parents as tutors, 10 min a day, 5 days a week for 14 weeks.
				Phon Aw	.19			
				RAN	.27			
		Follow-up mid 1st grade	30 (27)	Phon Aw	.09	Middle of first grade		
				Word reading	−.34			
		Follow-up end 1st grade		Phon Aw	−.11	End of first grade		
				Word reading	−.41			
*Note.* Many of the publications included in the review are based on data from the same sample or parts of the same sample. In a parenthesis in the first column of the tables, after the author names, a number in a parenthesis indicates which sample or part of a sample the data are based on. The samples are labelled as follows: Boets et al. = 1; Dutch sample of dyslexia = 2; Jyväskylä sample = 3; Danish sample = 4; Snowling et al. = 5; Pennington et al. = 6; Scarborough sample = 7; Smith et al. = 8; McBride-Chang et al. = 9; Hoeft et al. = 10; Gaab et al. = 11; de Bree et al. = 13; ELDEL sample = 14; Hindson et al. = 15. BAS = British Ability Scales; Bayley MDI = Bayley Mental Developmental Index; BNT = Boston Naming Test; BPVS = British Picture Vocabulary Scale; CELF = Clinical Evaluation of Language Fundamentals; CN Rep = Children’s Test of Nonword Repetition; Comp = comprehension; DAS = Differential Ability Scales; CPM = Colored Progressive Matrices; DEAP = Diagnostic Evaluation of Articulation and Phonology; GORT = Gray Oral Reading Tests; K-BIT = Kaufman Brief Intelligence Test; m-ABC = Movement Assessment Battery for Children; M CDI = MacArthur-Bates Communicative Development Inventories; NARA = Neale Analysis of Reading Ability; NEPSY = Developmental Neuropsychological Assessment; PA = phonological awareness; PCC = percentage consonants correct; Phon Aw = phonological awareness; Phon memory = phonological memory; PPVT = Peabody Picture Vocabulary Test; RAN = rapid naming; SON-R = Snijders-Oomen nonverbal intelligence tests; vSTM = verbal short-term memory; WAIS = Wechsler Adult Intelligence Scale; WPPSI = Wechsler Preschool and Primary Scale of Intelligence; WISC = Wechsler Intelligence Scale for Children; WJ = Woodcock Johnson; WOLD = Wechsler Objective Language Dimensions; WORD = Wechsler Objective Reading Dimensions; Word ID = word identification; WRMT= Woodcock Reading Mastery Tests; YARC = York Assessment of Reading for Comprehension.								
