# Supplementary material for: Oral Language Deficits in Familial Dyslexia: A Meta-Analysis and Review
Source: Psychol Bull. 2016 Jan 4;142(5):498–545. doi: 10.1037/bul0000037 (PMC4824243; doi:10.1037/bul0000037)
Supplement: Supplementary file 1 [file z2r006152529so1.doc]

**Supplemental Materials**

**Oral Language Deficits in Familial Dyslexia: A Meta-analysis and Review**

**by M. J.** **Snowling & M. Melby-Lervag, 2016, *Psychological Bulletin***

**http://dx.doi.org/10.1037/bul0000037**

Table S1

*Characteristics of Studies Using Neurophysiological Measures*

| Study name | Age level | Sample size | Method | Main results |
| --- | --- | --- | --- | --- |
| *Black, Tanaka, et al. 2012(10)* | 5–6 years | 51 (at risk compared with controls not at risk) | MRI | Found that maternal history of reading disability was associated with smaller bilateral prefrontal and parieto-temporal gray but not white matter results. Results remained unchanged when controlling for behavioral risk, SES and maternal education. No such risks were observed for paternal reading history. |
| *Guttorm, Leppanen, Hamalainen, et al. 2010 (3)* | 2.5–5 years  (longitudinal) | 49 (11 at risk compared with 10 controls not at risk) | ERP | Found that at-risk children with atypical speech processing in the right hemisphere (a slower shift in polarity from positivity to negativity in responses to /ga/ at 540–630 ms) scored significantly lower in phonological skills, rapid naming and letter knowledge than the control children. |
| *Guttorm, Leppanen, Tolvanen, et al. 2003*  *(3)* | Newborns | 49 (26 at risk compared with 23 controls not at risk) | ERP | Found that at-risk children differed significantly from control children in their response to /ga/ versus /ba/ and /da/in the right hemisphere at the latencies of 50–170 ms and 540–630 ms. The groups also differed in their responses to /da/ in the posterior electrode sites at 740–970 ms. The differences between the children at risk and the controls were robust across different methods of analysis. |
| *Guttorm, Leppanen, Richardson et al 2001(3)* | Newborns | 49 (26 at risk compared with 23 controls not at risk) | ERP | Analyses from averaged ERPs from latencies identified on the basis of principal component analyses showed significant group differences in stop–consonant processing in several latencies. The results indicate that the cortical electric activation differs between children with and without risk immediately after birth. |
| *Hosseini, Black, et al. 2013(10)* | 5.5 | 42 (22 at risk compared with 20 controls not at risk) | MRI | Results showed changes in topological properties known to be abnormal in dyslexia (left supramarginal gyrus, left inferior frontal gyrus) in beginning readers with a family risk. They also found changes in areas not associated with dyslexia but that can play a role in reading (left posterior cingulate, hippocampus and left precentral gyrus). |
| *Leppanen, Hamalainen, Guttorm, et al. 2011(3)* | Longitudinal (birth-school age) | (see Guttorm Leppanen, *et al.* 2003) | ERP | Found that early ERPs were correlated with kindergarten phonological processing and letter knowledge, and phoneme duration perception, reading and writing at school age. The correlations were in general more consistent among at risk readers, and at the group level, the ERPs differentiated between at-risk and control children. |
| *Leppanen, Hamalainen, Salminen, et al. 2010*  *(3)* | Longitudinal (birth-school age) | 8 reading disabled at risk, 14 fluent readers at risk and 25 controls not at risk | ERP | The brain responses of the typical readers with no family risk showed a clear differentiation between the tones varying in pitch and presented through an oddball paradigm, while the family-risk groups did not differentiate. The two at risk groups also showed a differential hemispheric ERP pattern. |
| *Leppanen, Richardson, Pihko, et al. 2002(3)* | 6 months | 37 at risk compared with 39 controls not at risk | ERP | The results showed that the infants at risk of dyslexia differed from control children both in their initial responsiveness to sounds per se and in their change detection responses dependent on the stimulus context. The results indicate that children with a family-risk process auditory temporal cues of speech sounds differently from children not at risk even before they learn to speak. |
| *Leppanen, Pihko, Eklund, et al. 1999(3)* | Newborns | 12 at risk compared with 11 controls not at risk | ERP | The results showed that responses to short deviant /ka/ were different from those to the long standard /kaa/ stimulus between children at risk and controls not at risk already in newborns. The results show that children at risk process speech/auditory stimuli different already at birth. |
| *Lovio, Naatanen & Kujala, 2010 (3)* | 6 years | 10 at risk compared with 9 controls not at risk | ERP (MMN) | The results showed that the amplitudes of the P1 responses elicited by the standard stimuli were smaller in the at risk group than in the control group. Also, the amplitudes of the mismatched negativity were smaller for the vowel, vowel-duration, consonants and intensity deviants in children at risk for dyslexia. The results are consistent with earlier studies reporting deviant auditory processing in children at risk for dyslexia. |
| *Maurer, Bucher, et al. 2003* | Kindergarten children | 31 at risk compared with 29 controls not at risk | ERP (MMN) | The results showed that during an early mismatch response segment, children at risk had more mid frontal positivity than controls, particularly in phoneme deviance. Significant group differences were found in the late MMN segment, where the mismatch response in children at risk was attenuated to frequency deviance and less left lateralized to phoneme deviance. The results suggest that children with a family risk have problems related to automatic processing of phoneme and simple tones. |
| *Noordenbos, Segers, et al. 2012* | 6 year old beginning readers | 31 at risk compared with 30 controls not at risk | MMN | The results showed that stimulus from different categories of phonemes elicited MMN in both groups, but that the MMN amplitude was clearly lower in the at risk children. The results also showed that the at risk children were sensitive to acoustic features that are irrelevant in their language. It is suggested that this sensitivity might hamper the development of stable phonological representations and can represent a cause in dyslexia. |
| *Pihko, Leppanen, Eklund, et al. 1999*  *(3)* | Newborn–6 months | 28 at risk compared with 23 controls not at risk | ERP | The ERPs of newborns had slow positive deflections typical for their age, but significant stimulus and group effects were found only by the age of 6 months. In both groups, the responses to the deviant /ka/ were more positive than responses to the standard /kaa/ stimuli. The results also suggest differences in brain activation patterns between the groups. |
| *Plakas, van Zuijen, van Leeuwen, et al. 2013(2)* | 41 months old–grade 2 | 30 at risk compared with 14 controls not at risk | ERP | The results showed that both auditory sensitivity to amplitude rise time (ART) and frequency processing were related to later reading skills. Also, independent of reading level, the at risk children in general showed impaired basic auditory processing, but it was not possible to discriminate between the at risk groups on the basis of auditory measures. The authors conclude that although related to reading disorders, the auditory ART and frequency measures lack the power to be considered as single cause predictors of dyslexia. |
| *Raschle, Stering, et al. 2013(11)* | 5:6 years (prereaders) | 14 at risk compared with 14 controls not at risk | fMRI | Results show functional alterations in left hemispheric regions during rapid auditory processing in prereading children, similar to those found in persons with dyslexia. Also, activation during rapid auditory processing correlates with prereading measures of phonological processing and with neural activation during phonological processing in posterior dorsal and ventral brain areas. |
| *Raschle, Zuk & Gaab, 2012(11)* | 66 months | 18 at risk compared with 18 controls not at risk | fMRI | The findings correspond with previously identified hypoactivations in left hemispheric posterior brain regions for school-aged children and adults with a diagnosis of dyslexia. Also, left occipitotemporal and temporoparietal brain activity is positively related to prereading skills in both groups. Results suggest that the differences in neural correlates is not a result of learning to read per se, but exists before reading starts. |
| *Raschle, Chang & Gaab, 2011 (11)* | 5:9 (pre readers) | 10 at risk compared with 10 controls not at risk | fMRI | Results showed significantly reduced gray matter volume indices in prereading children with a family risk of developmental dyslexia in left occipitotemporal, bilateral parietotemporal regions compared with children with no family risk. This also correlated with rapid naming. No differences between the groups were found in frontal and cerebellar regions. This suggests that differences in brain structure exist before reading starts. |
| *Regtvoort, van Leeuwen, et al. 2006(2)* | 5–8 year olds  (longitudinal) | 24 at risk compared with 14 controls not at risk | ERP | In this study, event-related potentials to visual standards were explored. The results showed an N1 habituation in the normal readers, but not in the normal reading at risk, and an N1 amplitude increase in the group of poor reading at risk and poor reading controls. No P3 habituation was shown in either of the groups. |
| *Torkildsen, Syversen, et al. 2007* | 20–24 months | 9 at risk compared with 27 controls not at risk | ERP | Two experiments showed that an early component assumed to reflect facilitated lexical processing for primed words was enhanced in the at risk group compared with the control group. Also, a N400-like response, which was prominent in the control group, was not present in the at risk group. Results reflect that efficiencies in young children at risk of dyslexia are not restricted only to perceptual or lower level phonological abilities, but also present in higher order language skills such as semantic processing. |
| *Van Herten, Pasman, et al. 2008(2)* | 17 months | 60 at risk compared with 48 controls not at risk | AERP | In this study, AERPs were elicited by standard stimuli (/bAk/) and deviant (/dAk/) word stimuli presented in an oddball paradigm. The at risk children tended to show delayed P1 and P2 peaks for standard stimuli. Hemispheric group differences were found for N2 amplitude and P1 latency, suggesting an atypical processing of words in the at risk children. |
| *Van Leeuwen, Been, et al. 2007 (2)* | 2 months | 32 at risk compared with 18 controls not at risk | ERP | In this study, manipulated (/bAk/) and (/dAk/) speech stimuli were used as frequently presented standards. The neuroelectric brain responses of the control children were highly sensitive to the phoneme boundary that separated these stimuli, whilst the at risk children were not. Sources of responses were mainly found in the left hemisphere for the control children, but in the right hemisphere for children at risk. This suggests that problems in children with dyslexia are present long before the onset of reading instruction. |
| *Van Zuijen, Plakas, et al. 2013(2)* | 2 months–2^nd^ grade (longitudinal) | 26 at risk compared with 12 controls not at risk | ERP | This study examined whether the auditory system processes /bAk/ and /dAk/ differently in children at risk versus controls. The results showed that the fluent-reading children from both groups processed the speech sound changes differently in infancy as indicated by a mismatched response. In the control group, the MMR was frontally positive, while in the at risk group, it was parietally positive. The results indicate a very early speech processing deficit in children that later becomes nonfluent readers. |
| *Van Leeuwen, Been, et al. 2008 (2)* | 2 months | 82 at risk compared with 57 controls not at risk | ERP | This study examined whether the auditory system processes /bAk/ and /dAk/ differently in children at risk versus controls. The results showed that infants at risk had an attenuated early mismatched response and an absent late one, in addition to diminished cortical activity in the left hemisphere. The results are consistent with a temporal processing deficit in the infants at risk and may reflect an early precursor of the disorder. |
